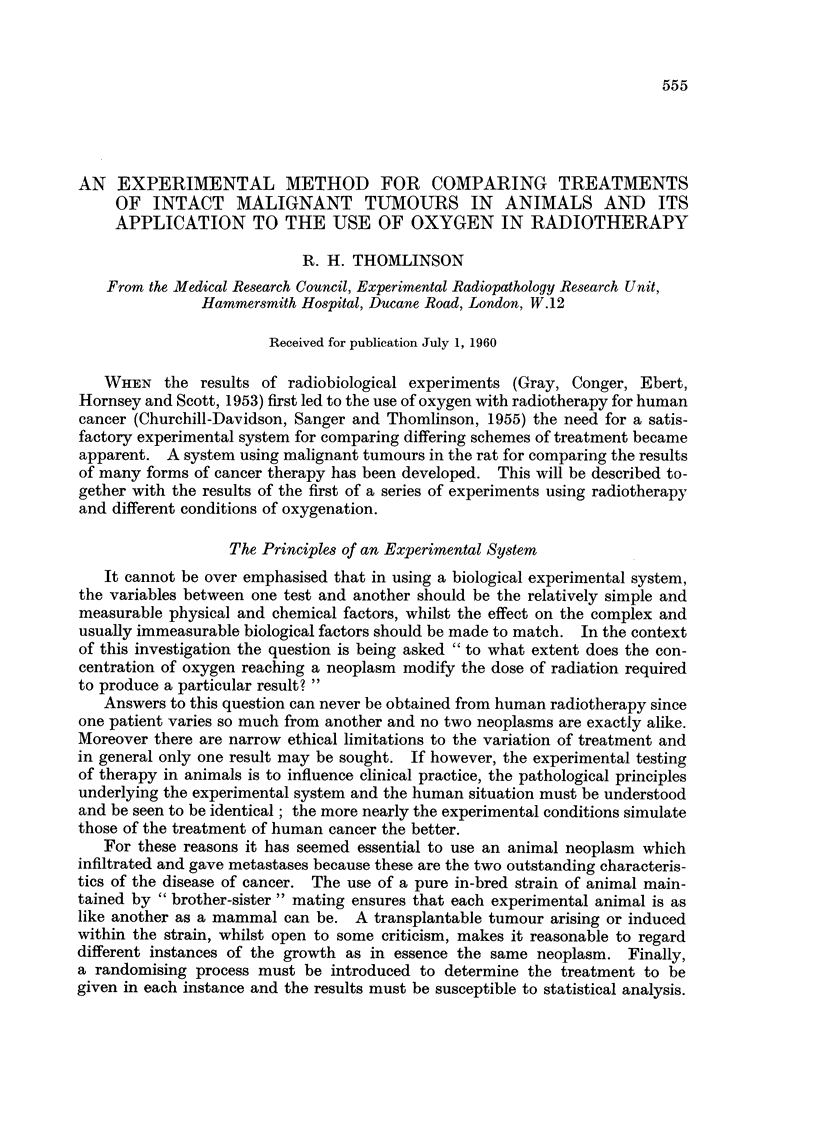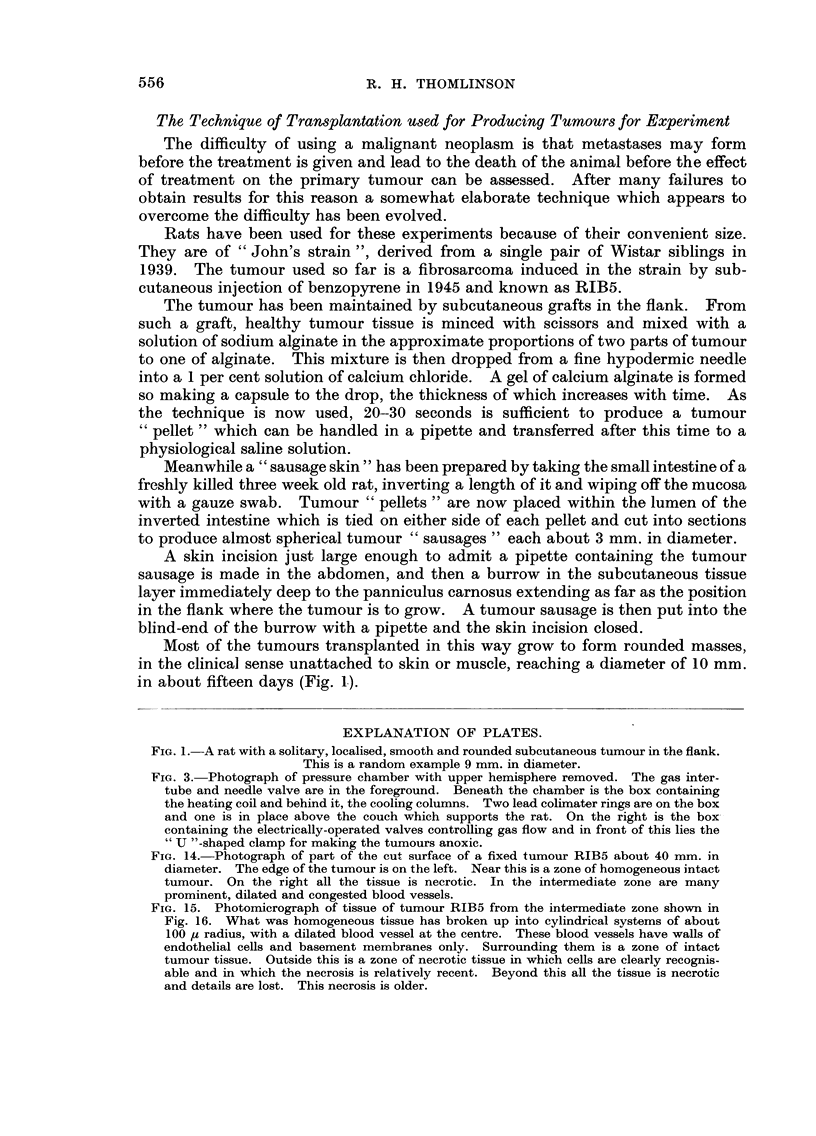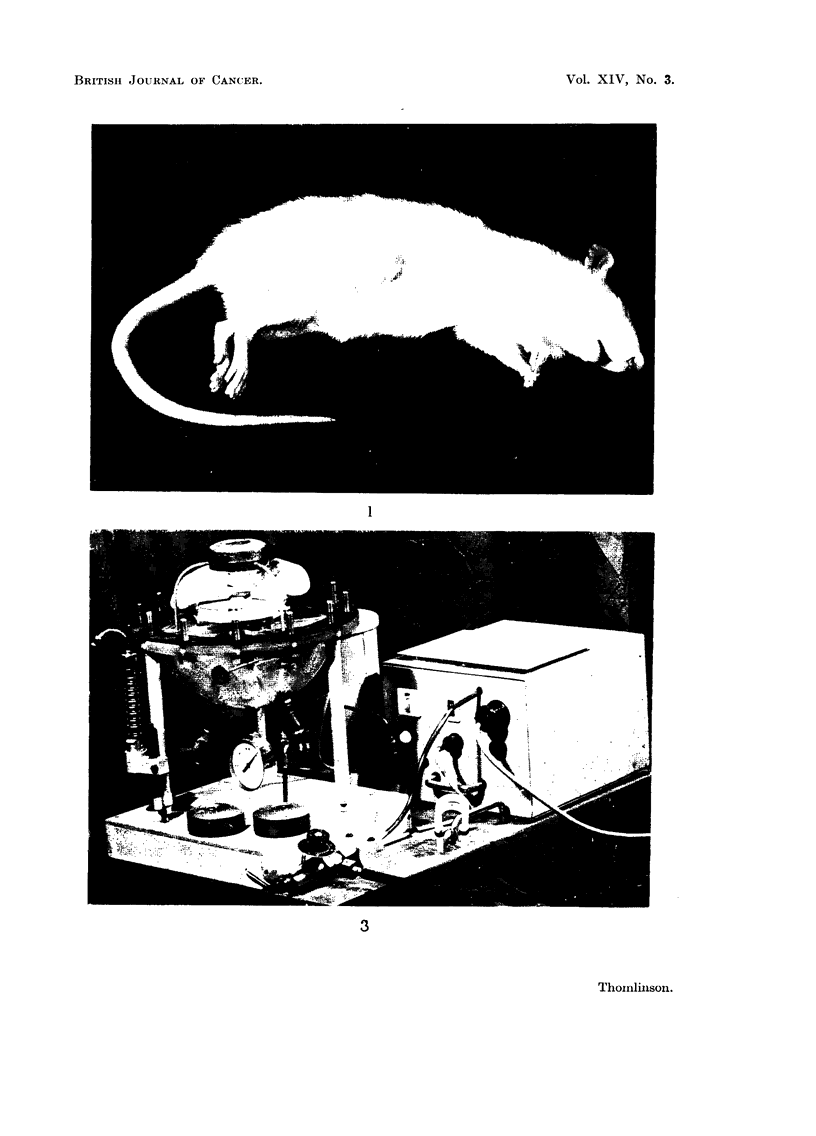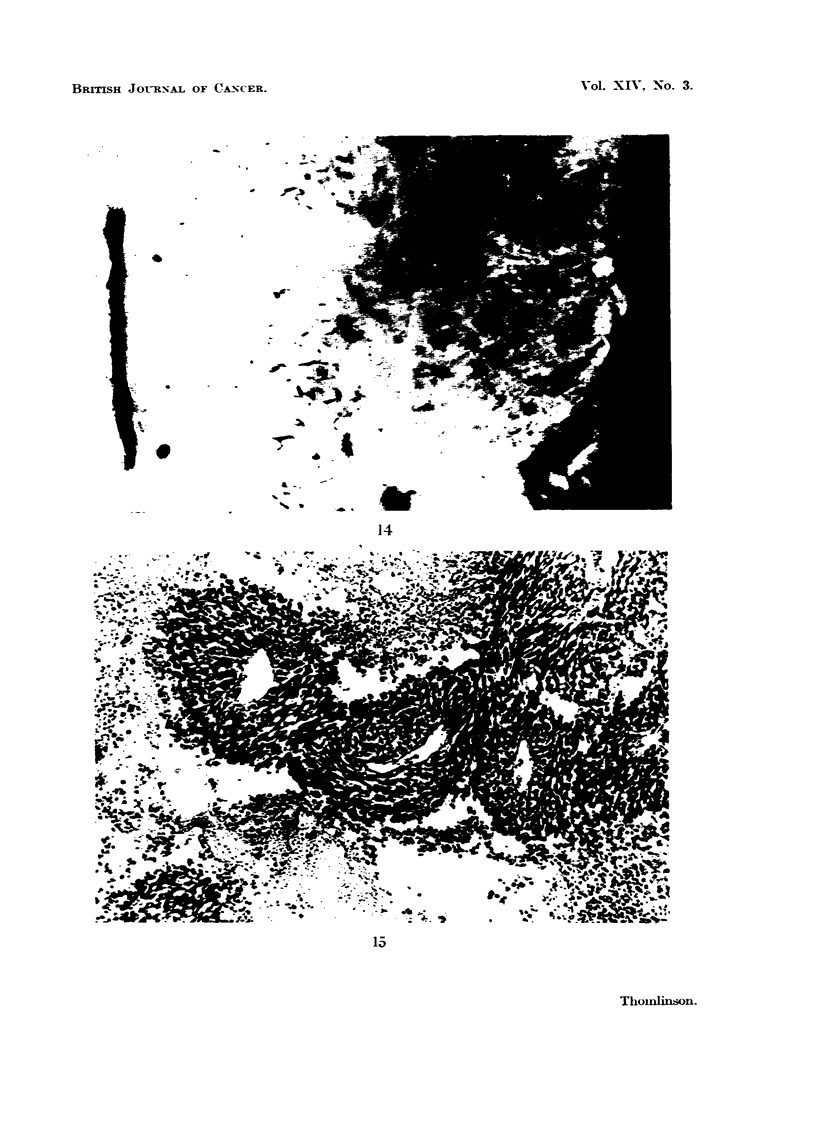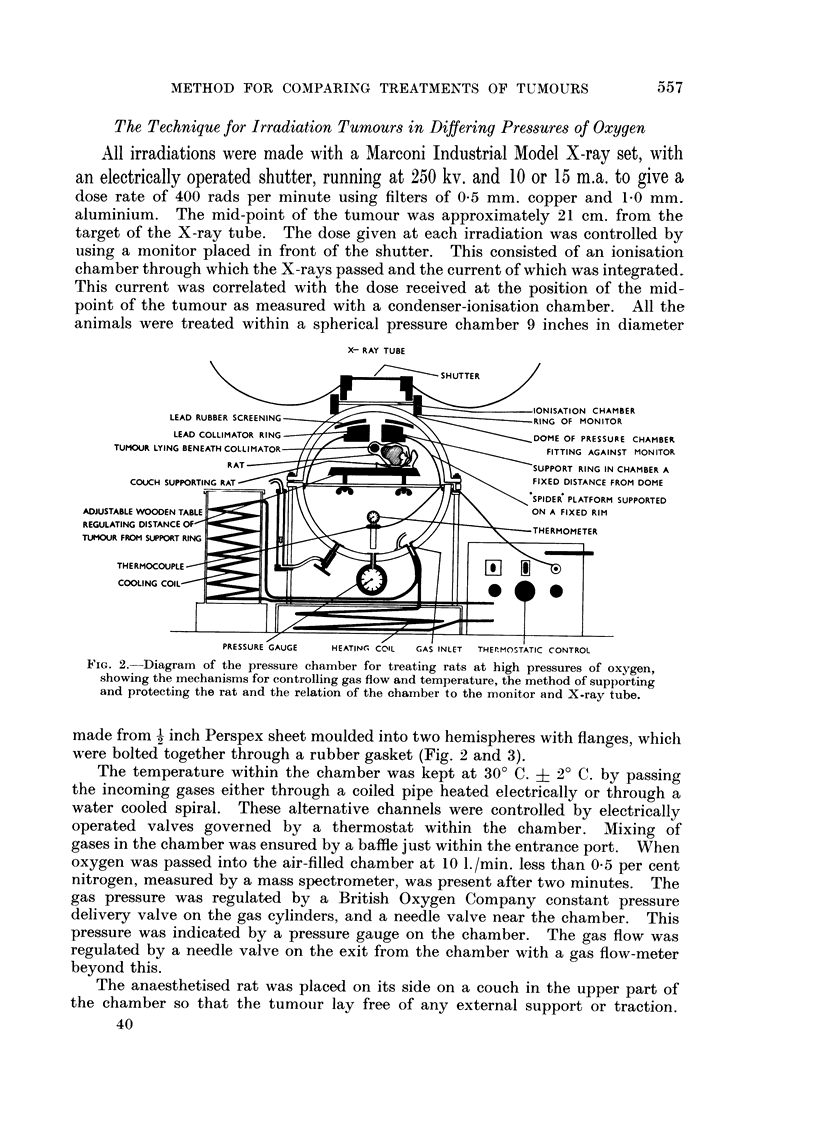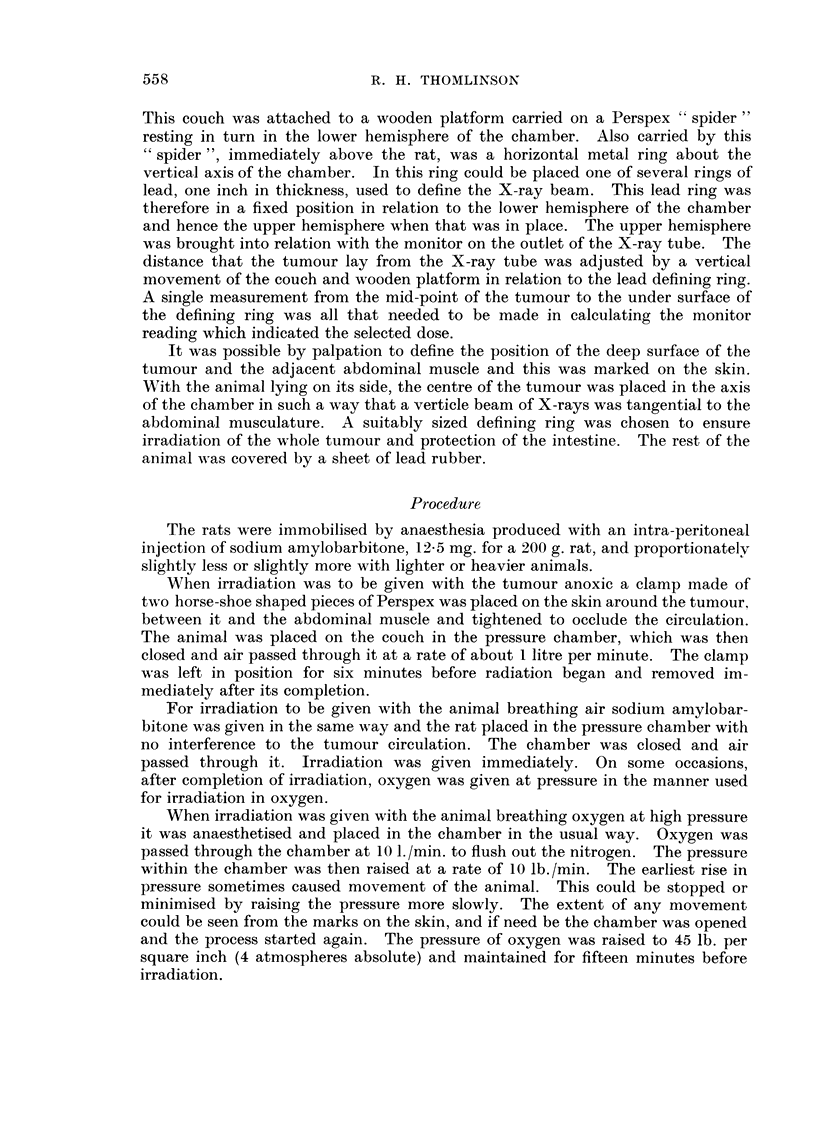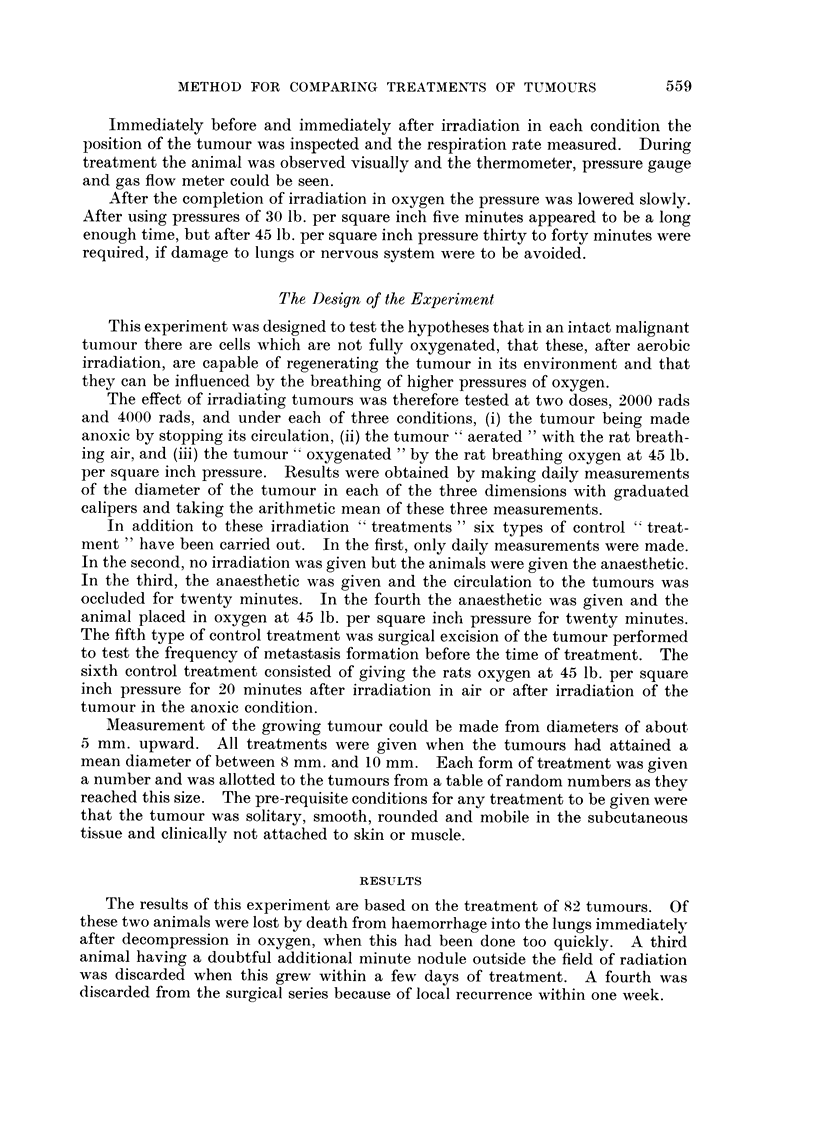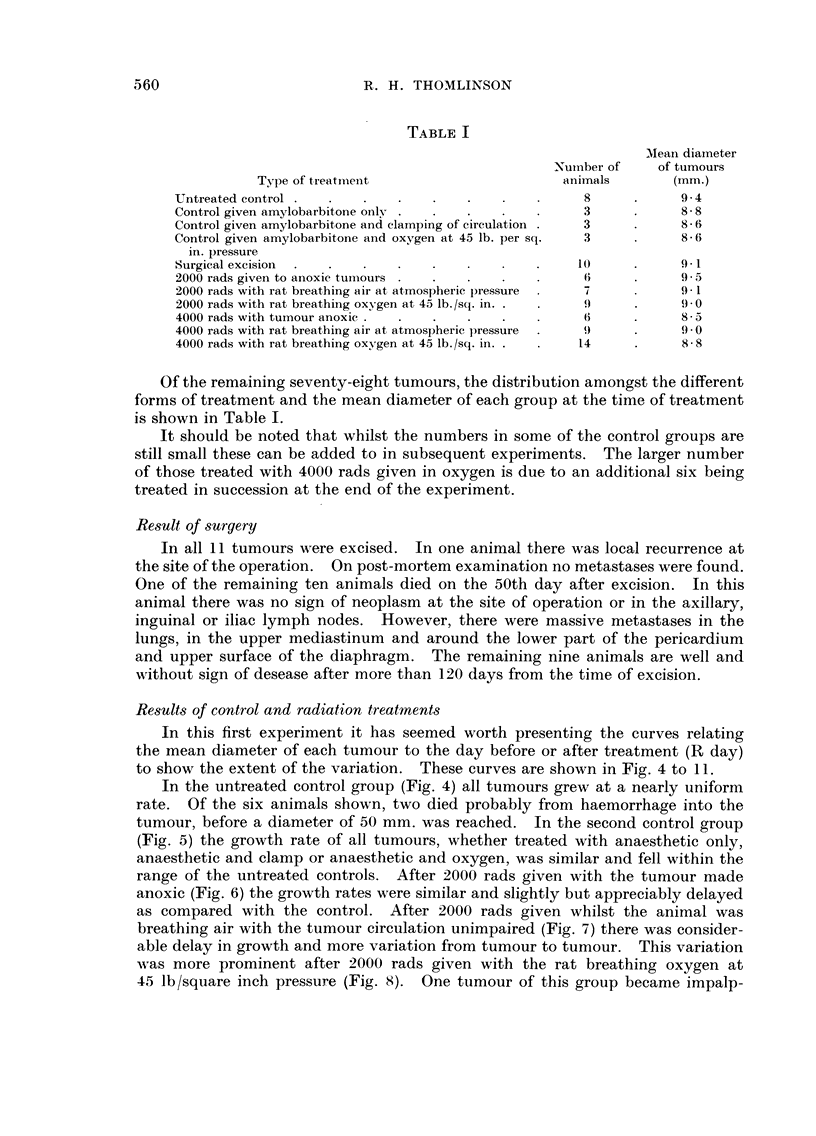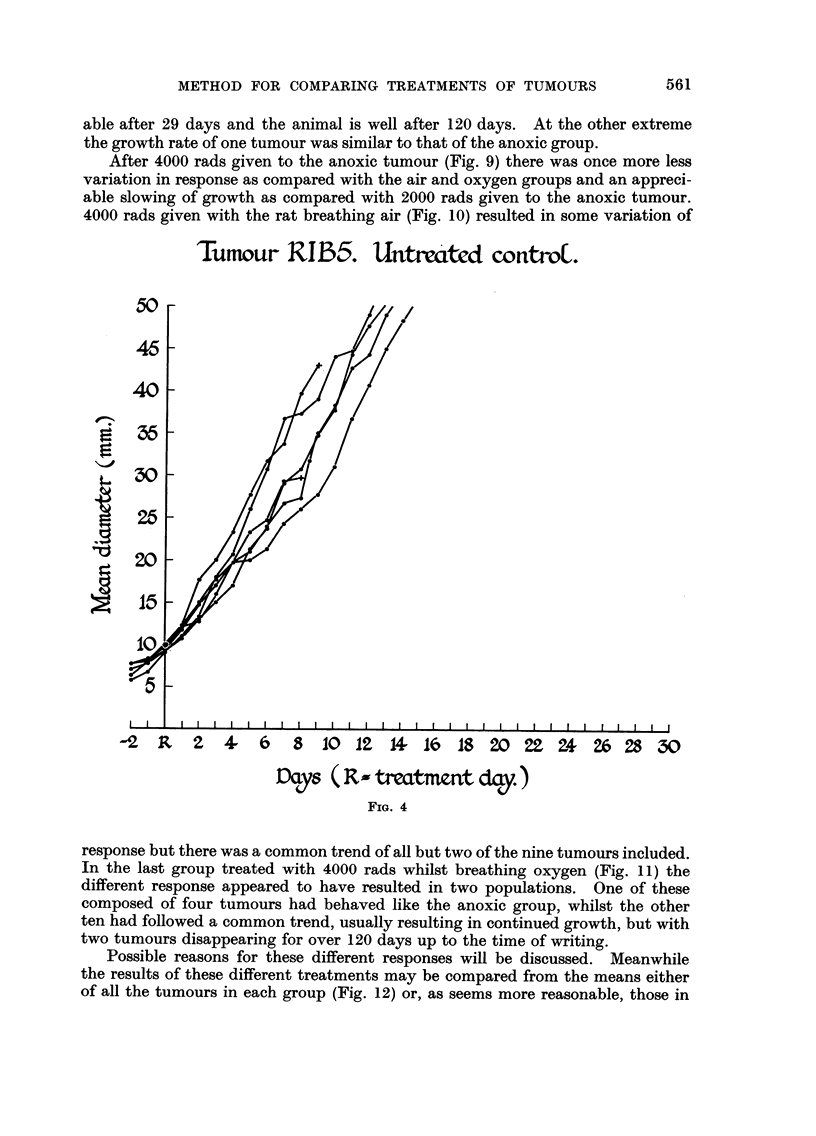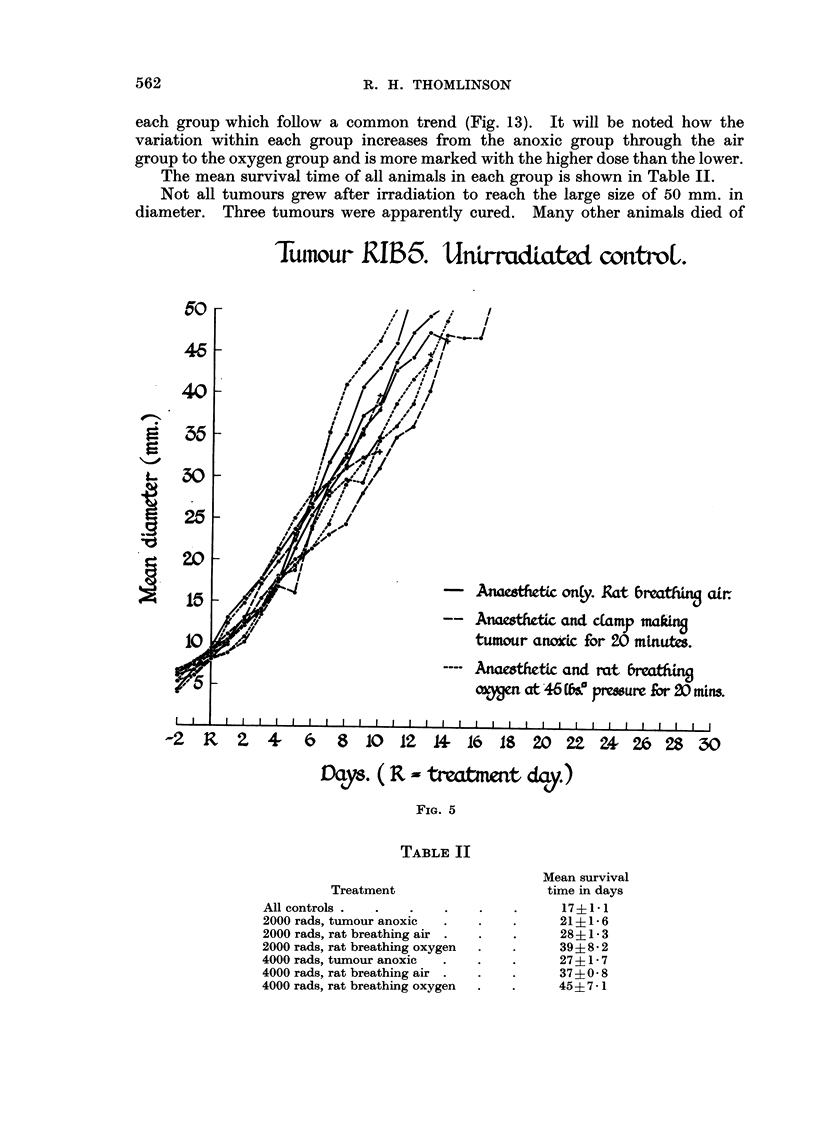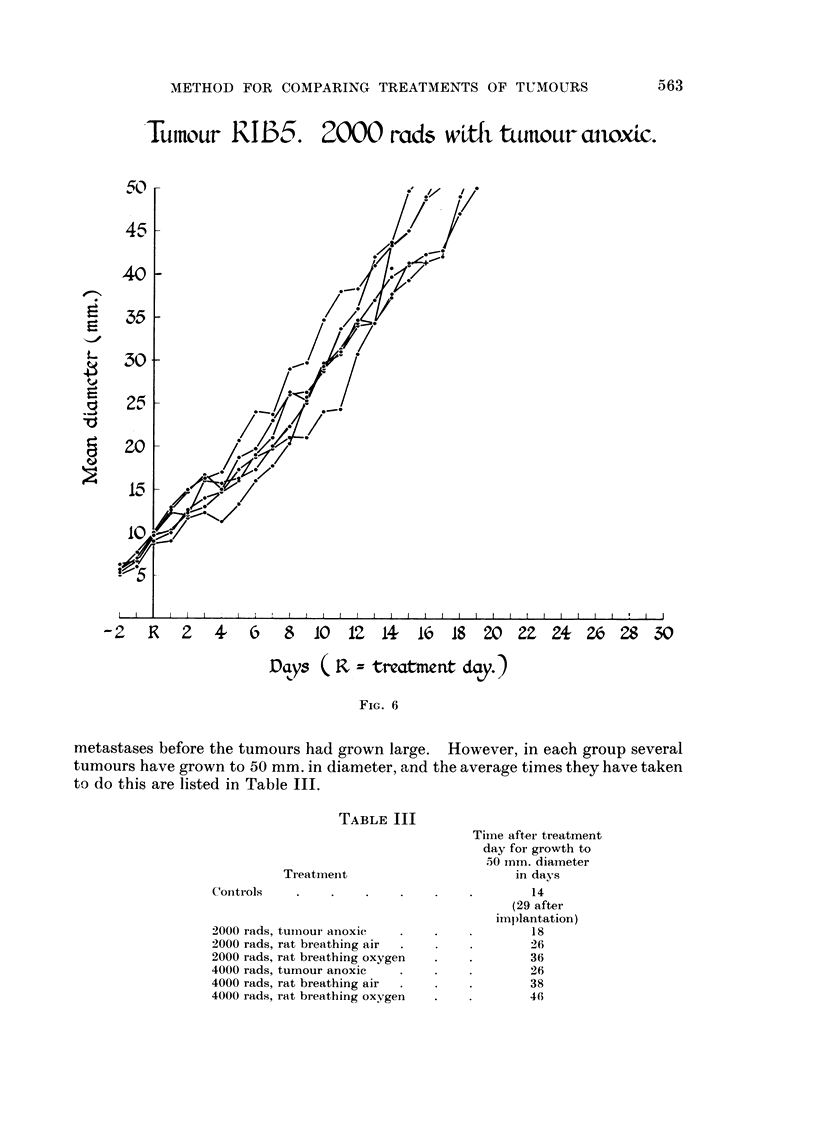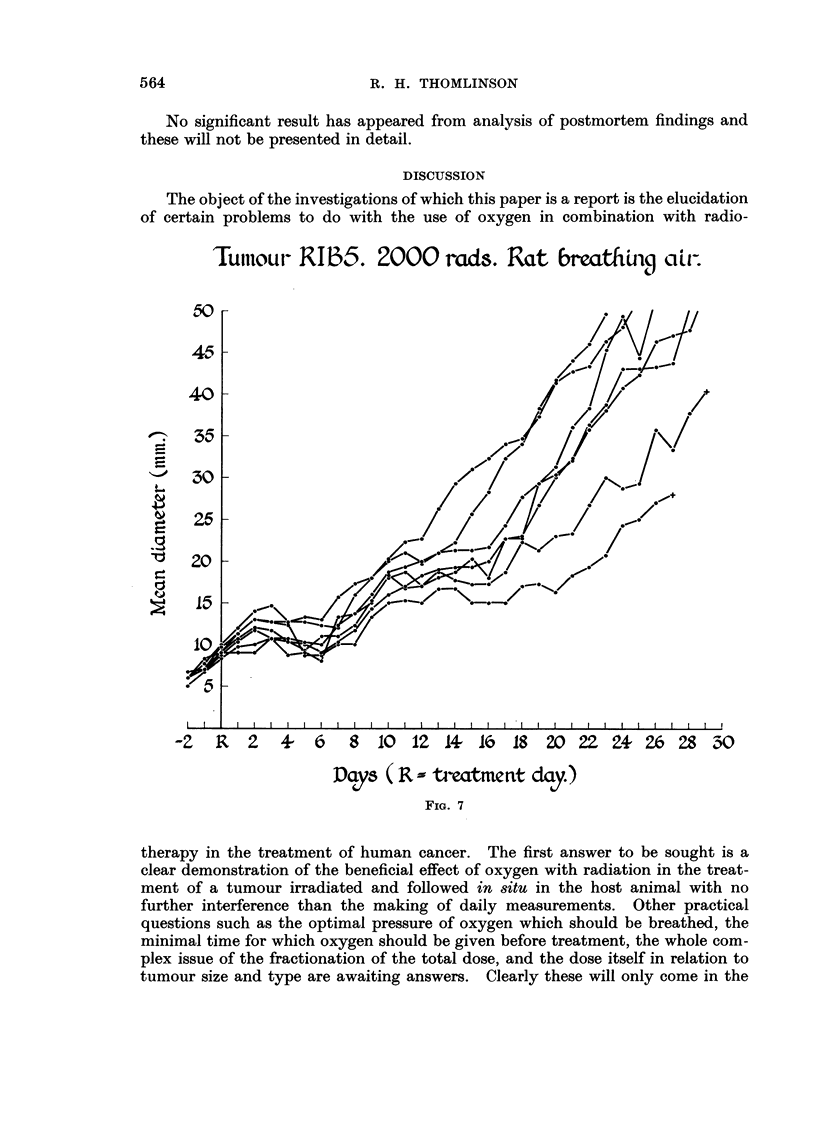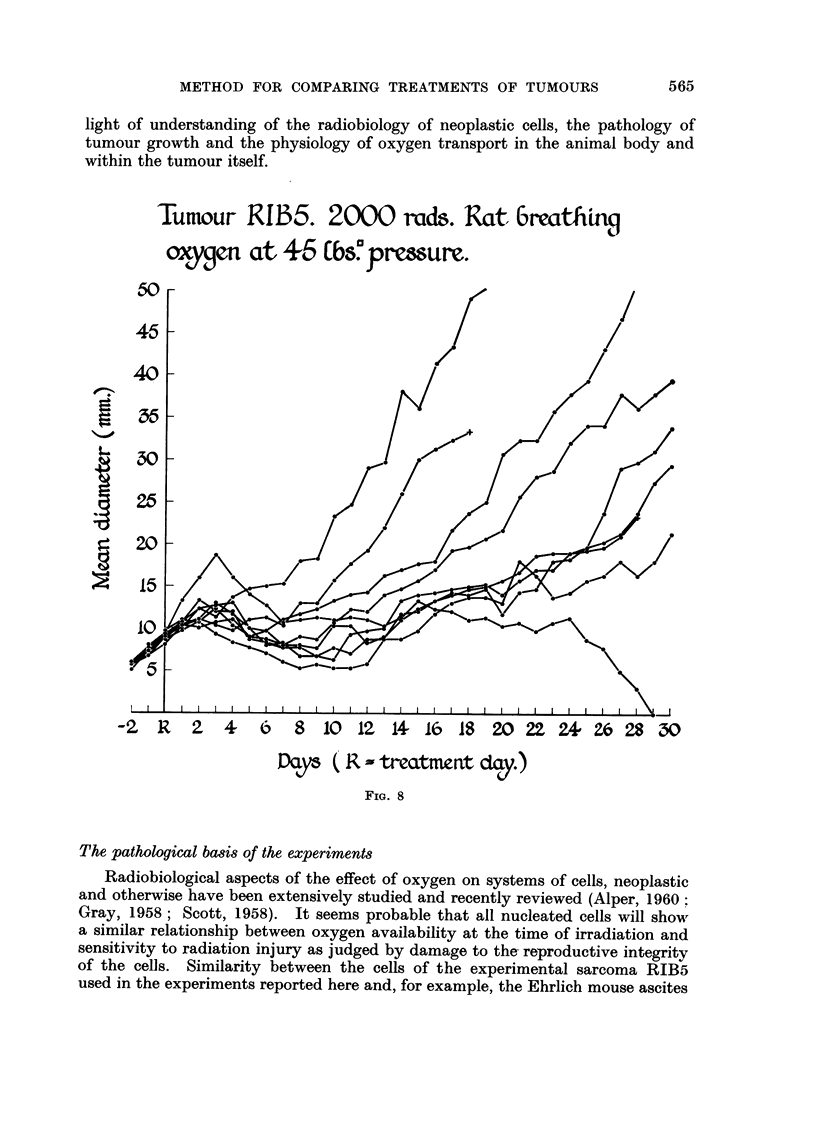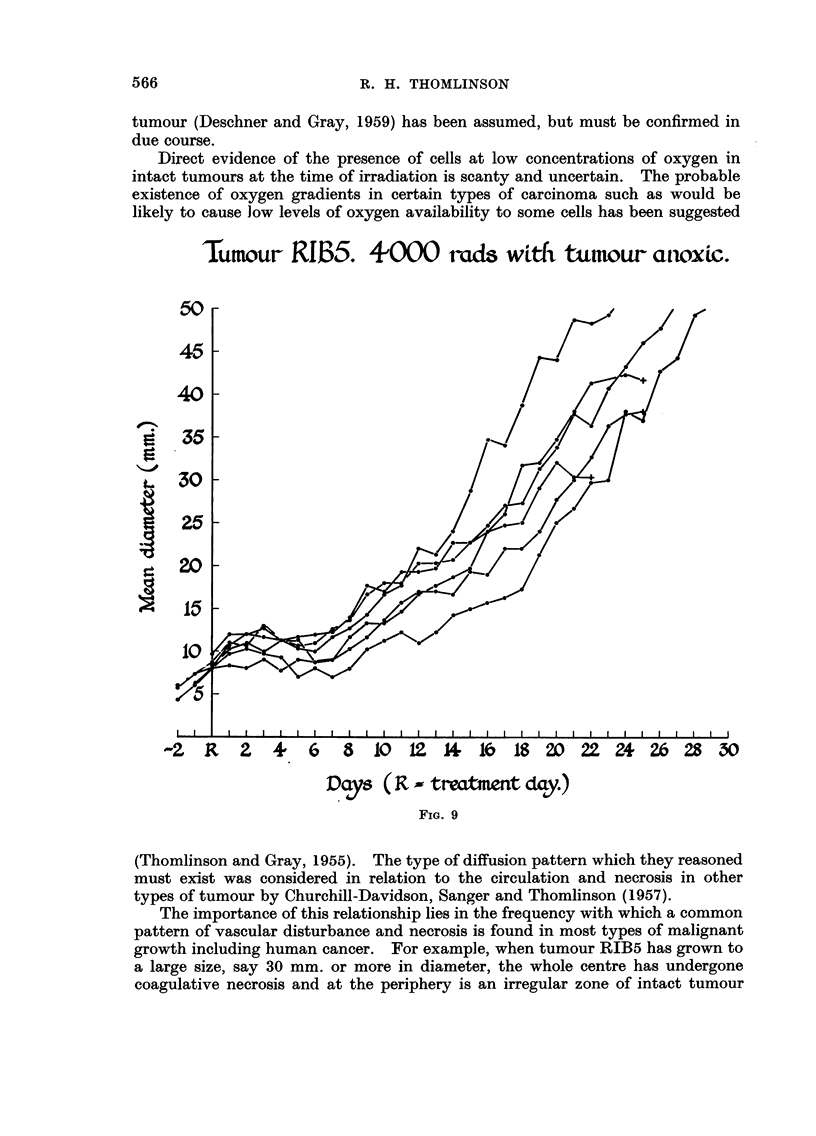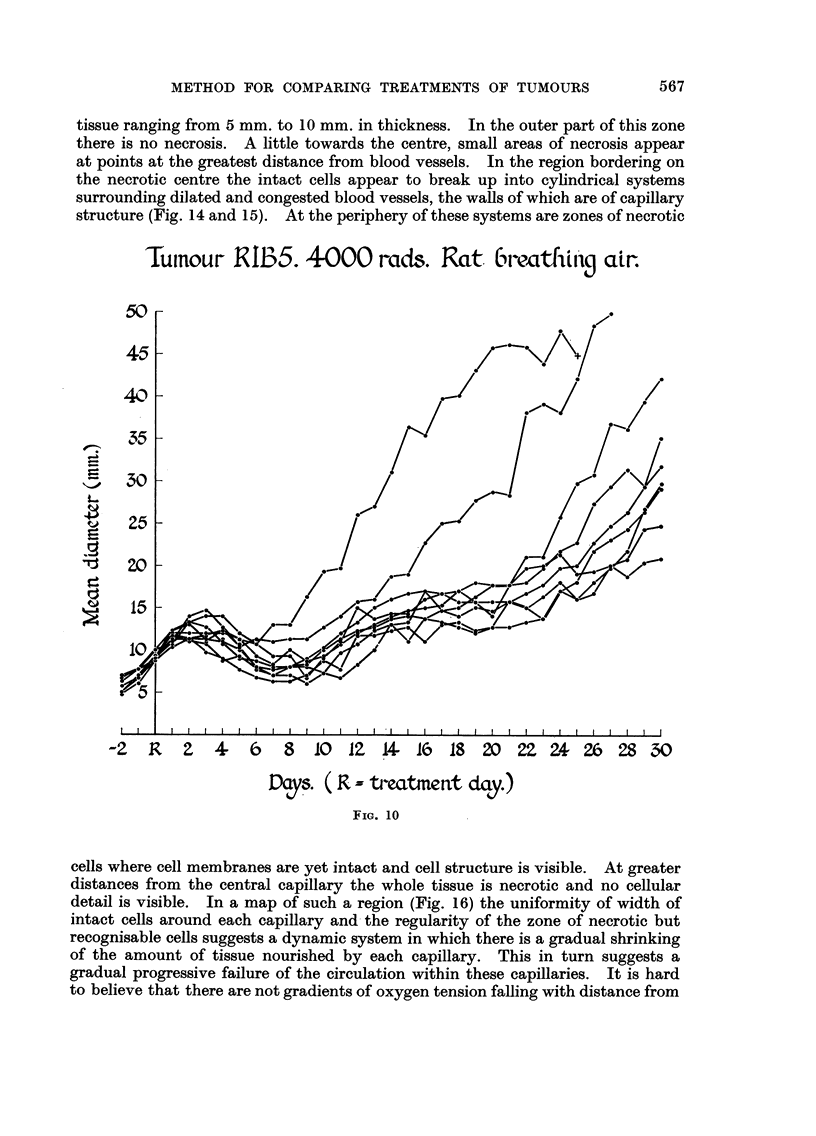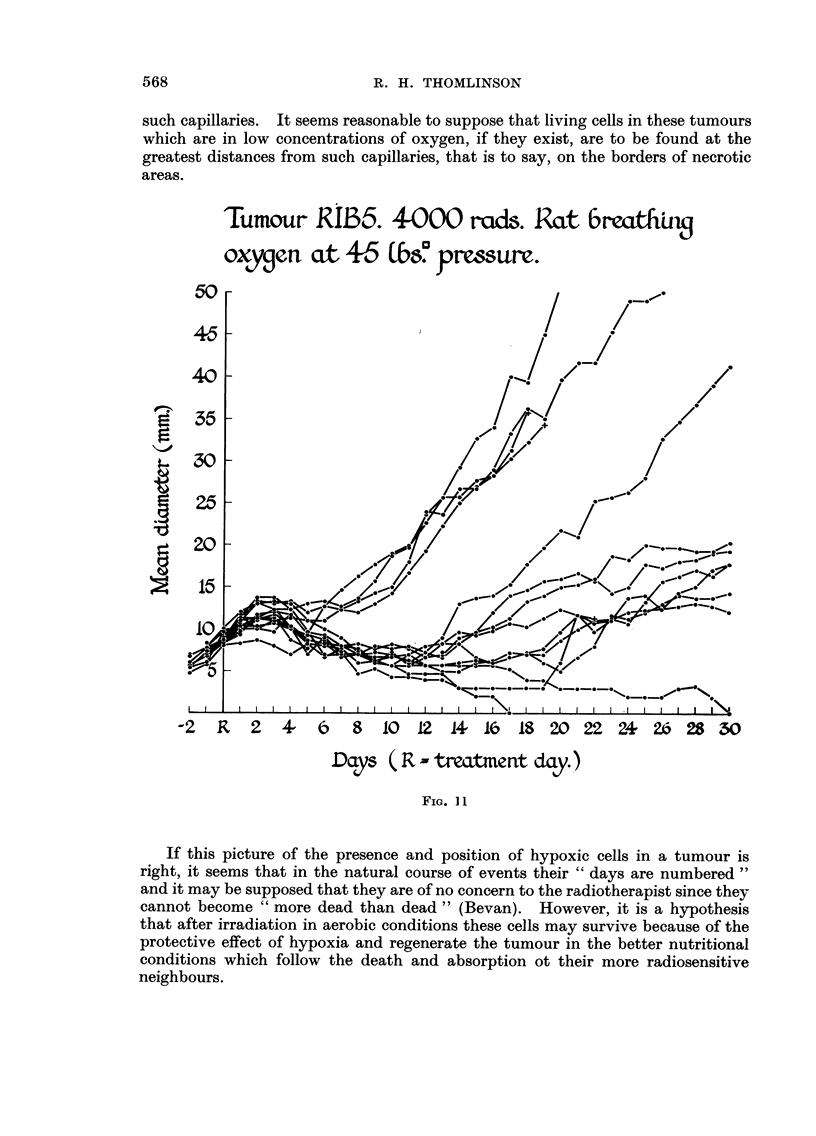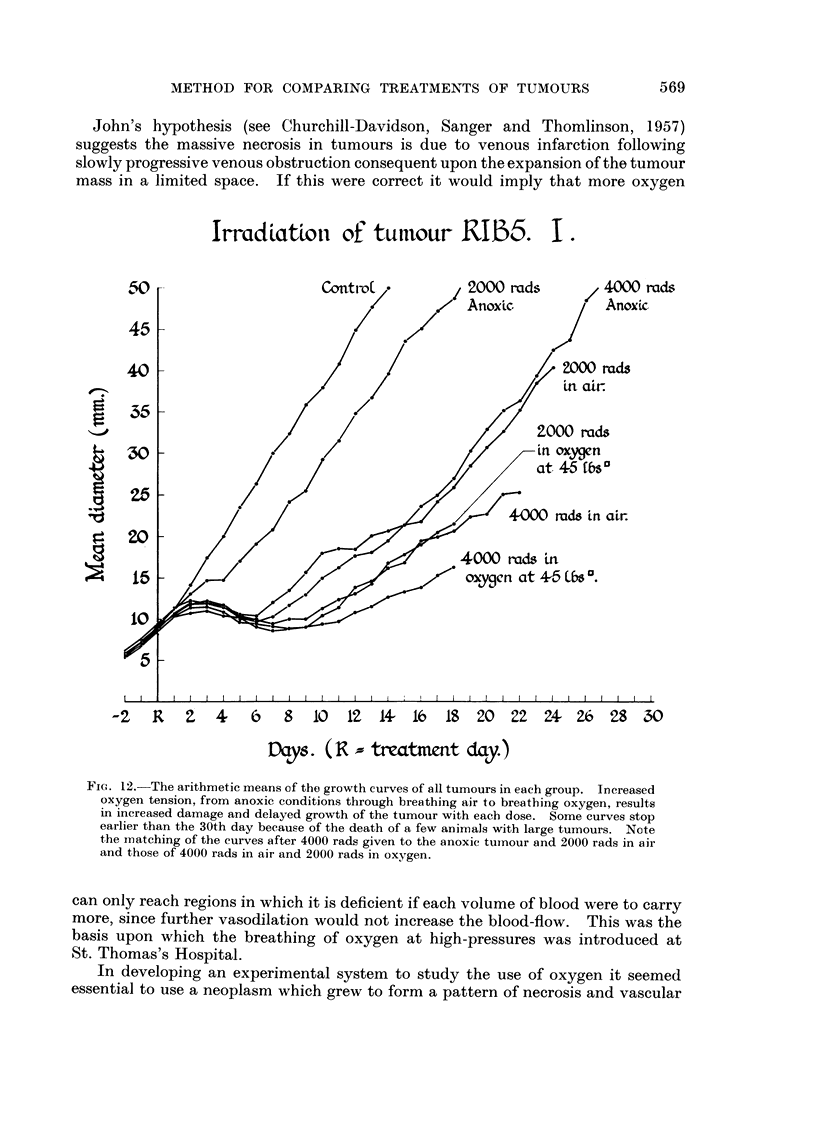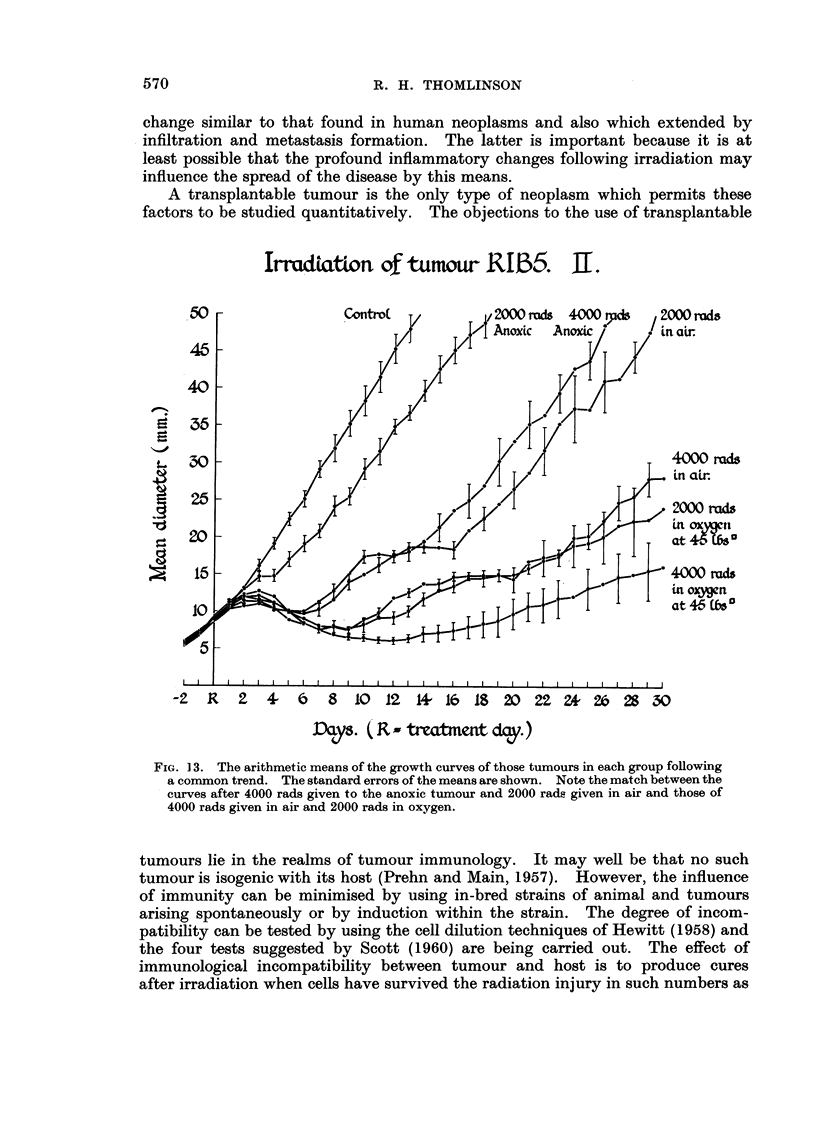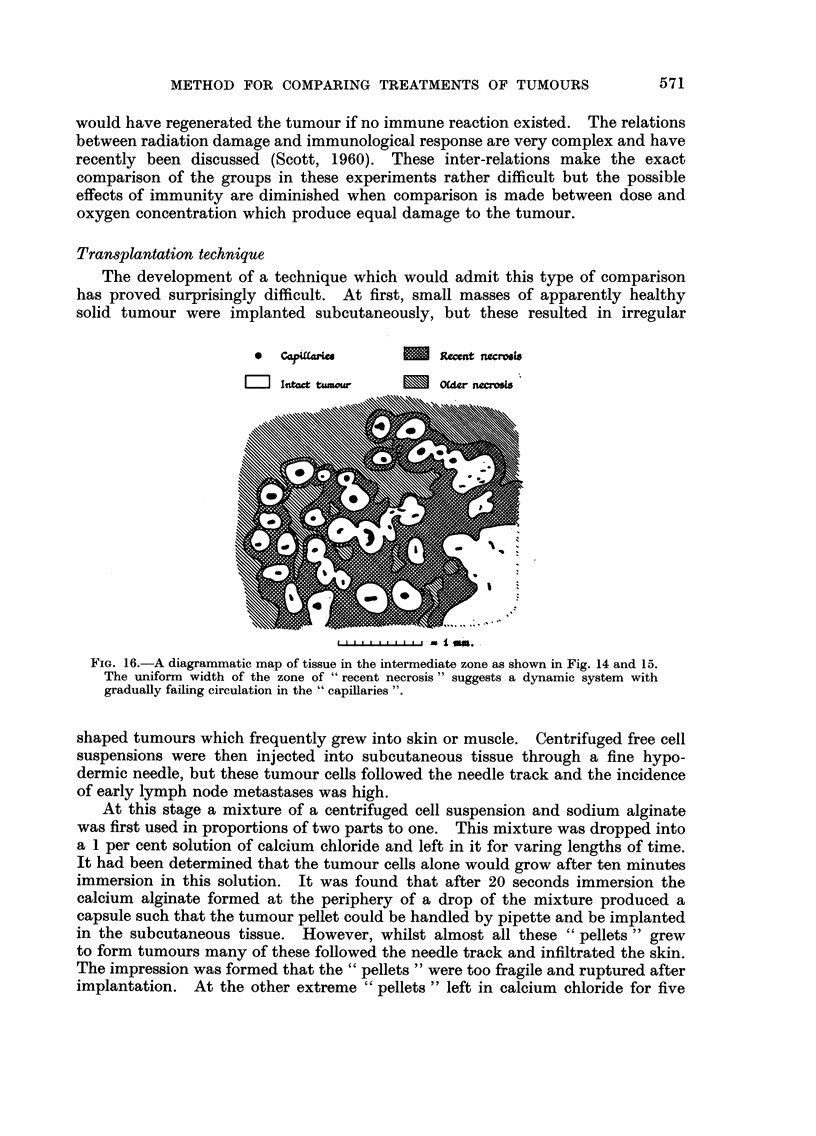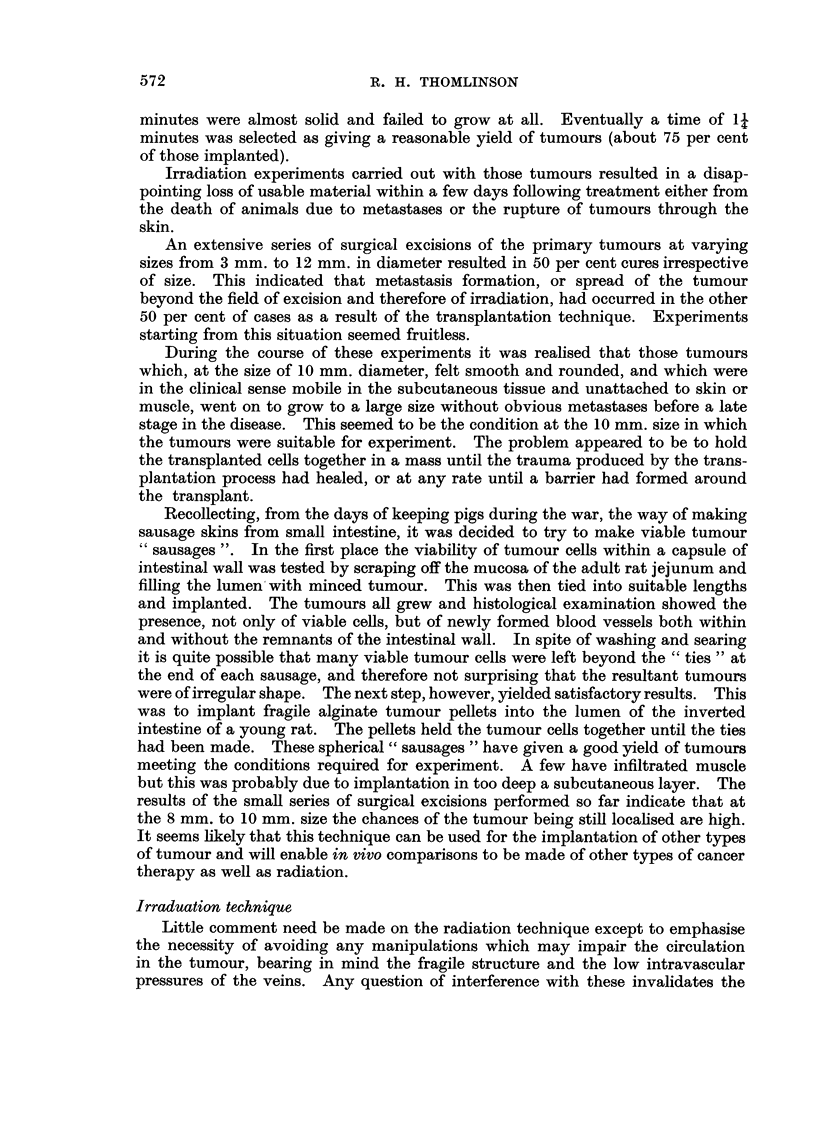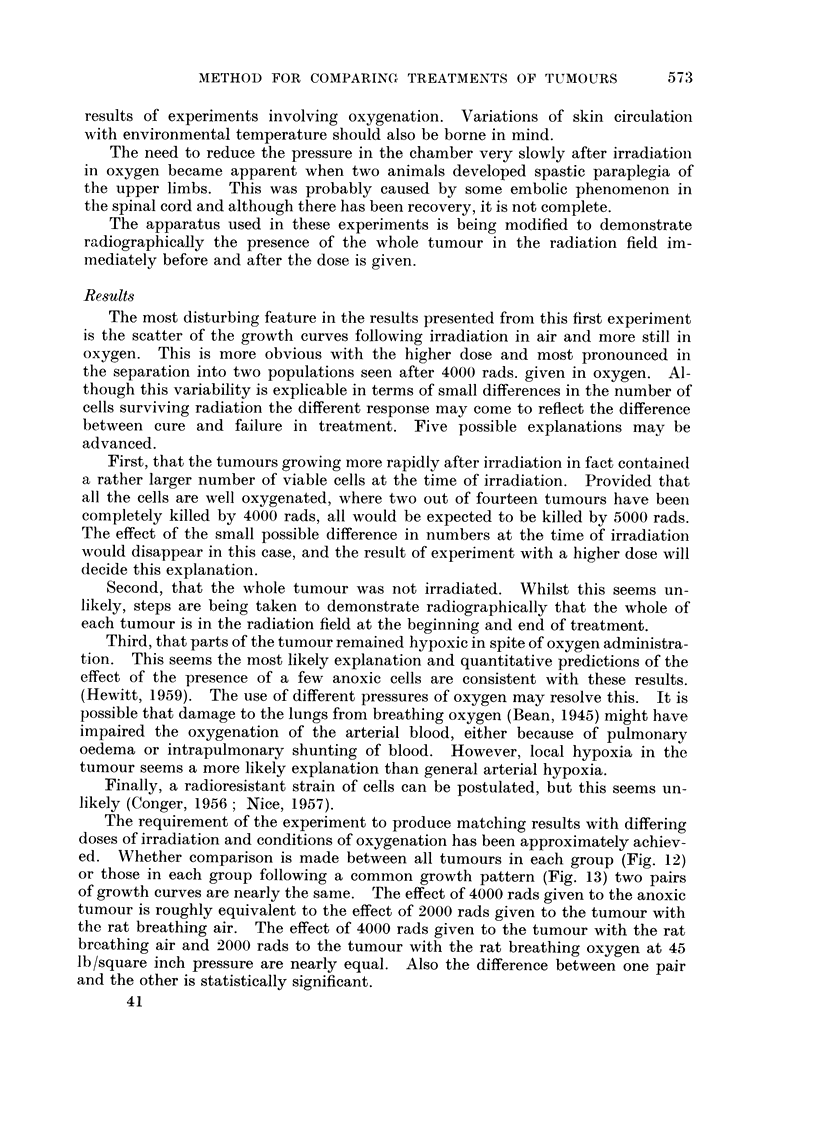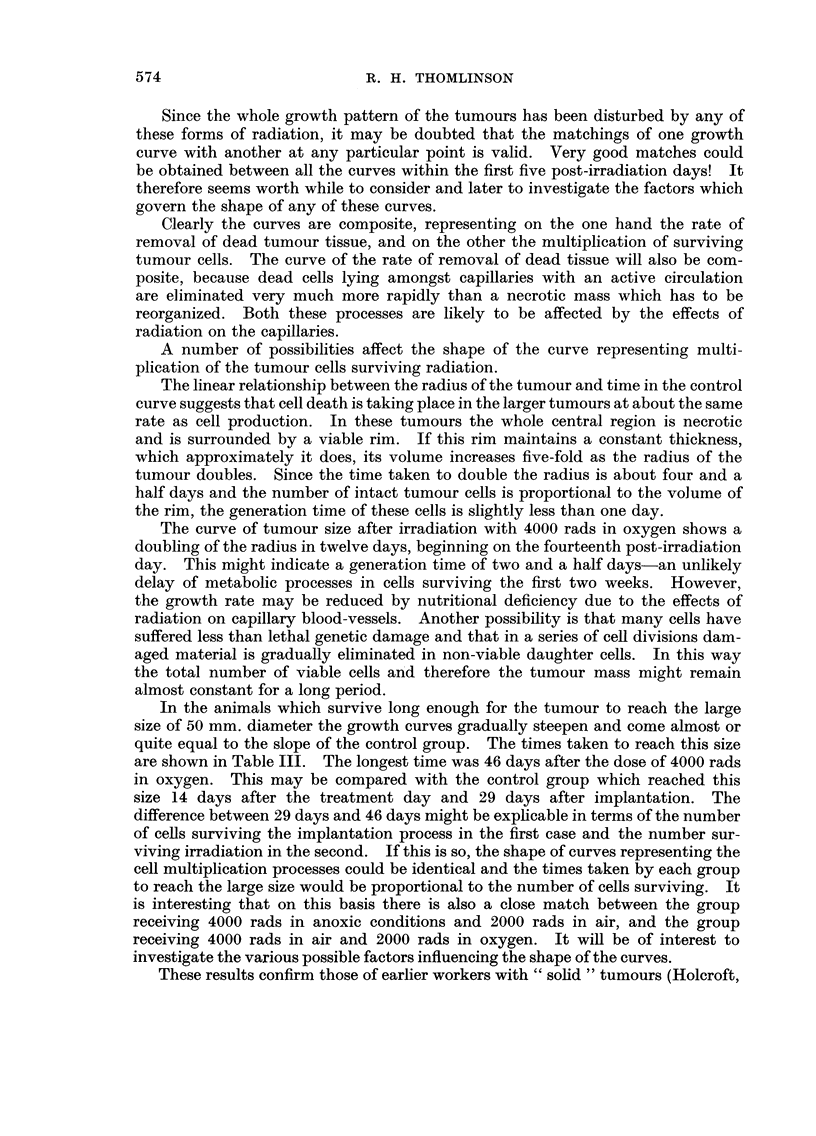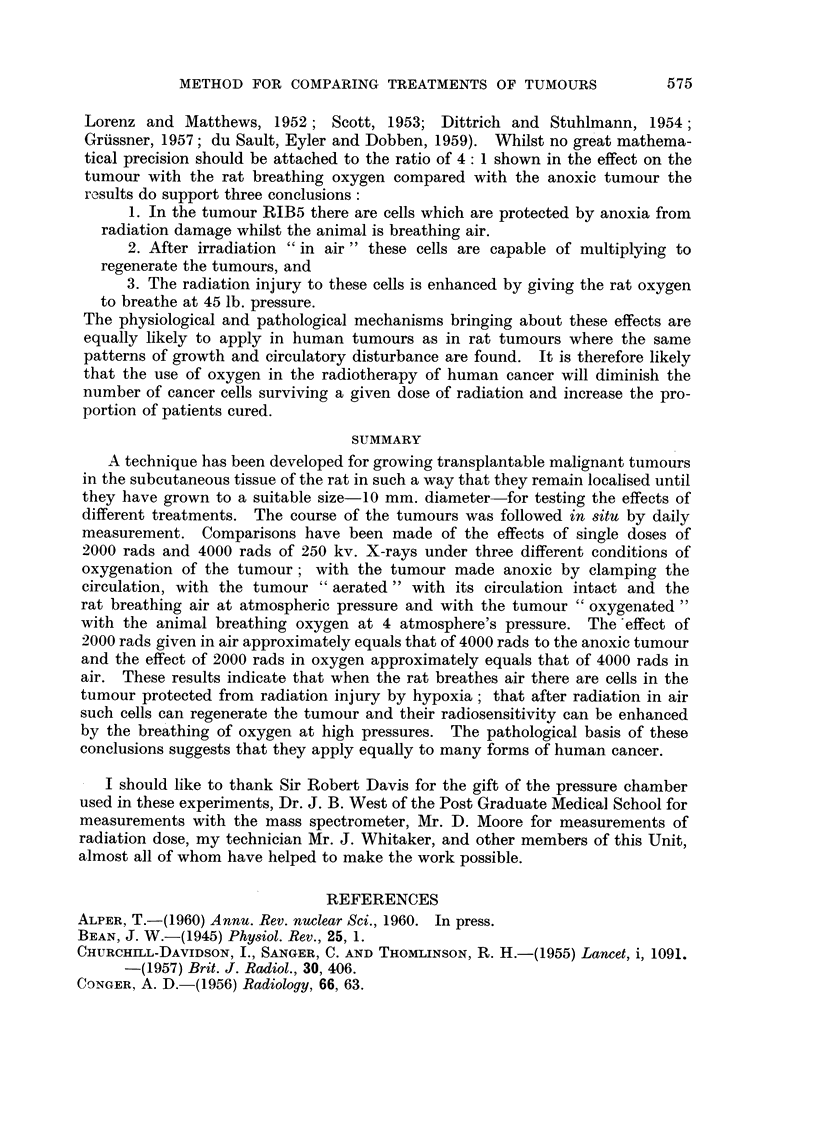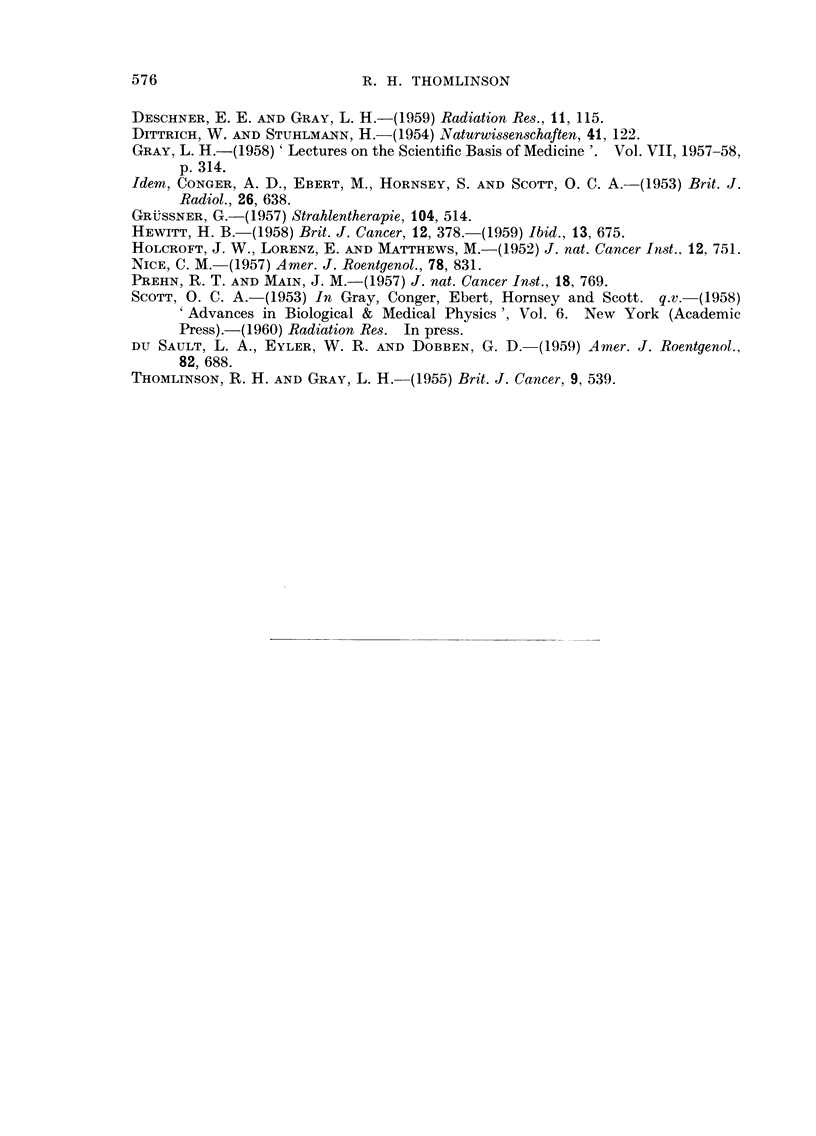# An Experimental Method for Comparing Treatments of Intact Malignant Tumours in Animals and its Application to the Use of Oxygen in Radiotherapy

**DOI:** 10.1038/bjc.1960.62

**Published:** 1960-09

**Authors:** R. H. Thomlinson

## Abstract

**Images:**


### AN EXPERIMENTAL METHOD FOR COMPARING TREATMENTS OF INTACT MALIGNANT TUMOURS IN ANIMALS AND ITS APPLICATION TO THE USE OF OXYGEN IN RADIOTHERAPY

R. H. THOMLINSON

*From the Medical Research Council, Experimental Radiopathology Research Unit, Hammersmith Hospital, Ducane Road, London, W.12*

Received for publication July 1, 1960

WHEN the results of radiobiological experiments (Gray, Conger, Ebert, Hornsey and Scott, 1953) first led to the use of oxygen with radiotherapy for human cancer (Churchill-Davidson, Sanger and Thomlinson, 1955) the need for a satisfactory experimental system for comparing differing schemes of treatment became apparent. A system using malignant tumours in the rat for comparing the results of many forms of cancer therapy has been developed. This will be described together with the results of the first of a series of experiments using radiotherapy and different conditions of oxygenation.

### *The Principles of an Experimental System*

It cannot be over emphasised that in using a biological experimental system, the variables between one test and another should be the relatively simple and measurable physical and chemical factors, whilst the effect on the complex and usually immeasurable biological factors should be made to match. In the context of this investigation the question is being asked " to what extent does the concentration of oxygen reaching a neoplasm modify the dose of radiation required to produce a particular result? "

Answers to this question can never be obtained from human radiotherapy since one patient varies so much from another and no two neoplasms are exactly alike. Moreover there are narrow ethical limitations to the variation of treatment and in general only one result may be sought. If however, the experimental testing of therapy in animals is to influence clinical practice, the pathological principles underlying the experimental system and the human situation must be understood and be seen to be identical ; the more nearly the experimental conditions simulate those of the treatment of human cancer the better.

For these reasons it has seemed essential to use an animal neoplasm which infiltrated and gave metastases because these are the two outstanding characteristics of the disease of cancer. The use of a pure in-bred strain of animal maintained by " brother-sister " mating ensures that each experimental animal is as like another as a mammal can be. A transplantable tumour arising or induced within the strain, whilst open to some criticism, makes it reasonable to regard different instances of the growth as in essence the same neoplasm. Finally, a randomising process must be introduced to determine the treatment to be given in each instance and the results must be susceptible to statistical analysis.

*The Technique of Transplantation used for Producing Tumours for Experiment*

The difficulty of using a malignant neoplasm is that metastases may form before the treatment is given and lead to the death of the animal before the effect of treatment on the primary tumour can be assessed. After many failures to obtain results for this reason a somewhat elaborate technique which appears to overcome the difficulty has been evolved.

Rats have been used for these experiments because of their convenient size. They are of " John's strain ", derived from a single pair of Wistar siblings in 1939. The tumour used so far is a fibrosarcoma induced in the strain by subcutaneous injection of benzopyrene in 1945 and known as RIB5.

The tumour has been maintained by subcutaneous grafts in the flank. From such a graft, healthy tumour tissue is minced with scissors and mixed with a solution of sodium alginate in the approximate proportions of two parts of tumour to one of alginate. This mixture is then dropped from a fine hypodermic needle into a 1 per cent solution of calcium chloride. A gel of calcium alginate is formed so making a capsule to the drop, the thickness of which increases with time. As the technique is now used, 20–30 seconds is sufficient to produce a tumour " pellet " which can be handled in a pipette and transferred after this time to a physiological saline solution.

Meanwhile a " sausage skin " has been prepared by taking the small intestine of a freshly killed three week old rat, inverting a length of it and wiping off the mucosa with a gauze swab. Tumour " pellets " are now placed within the lumen of the inverted intestine which is tied on either side of each pellet and cut into sections to produce almost spherical tumour " sausages " each about 3 mm. in diameter.

A skin incision just large enough to admit a pipette containing the tumour sausage is made in the abdomen, and then a burrow in the subcutaneous tissue layer immediately deep to the panniculus carnosus extending as far as the position in the flank where the tumour is to grow. A tumour sausage is then put into the blind-end of the burrow with a pipette and the skin incision closed.

Most of the tumours transplanted in this way grow to form rounded masses, in the clinical sense unattached to skin or muscle, reaching a diameter of 10 mm. in about fifteen days (Fig. 1).

---

EXPLANATION OF PLATES.

FIG. 1.—A rat with a solitary, localised, smooth and rounded subcutaneous tumour in the flank. This is a random example 9 mm. in diameter.

FIG. 3.—Photograph of pressure chamber with upper hemisphere removed. The gas inter-tube and needle valve are in the foreground. Beneath the chamber is the box containing the heating coil and behind it, the cooling columns. Two lead colimater rings are on the box and one is in place above the couch which supports the rat. On the right is the box containing the electrically-operated valves controlling gas flow and in front of this lies the " U "-shaped clamp for making the tumours anoxic.

FIG. 14.—Photograph of part of the cut surface of a fixed tumour RIB5 about 40 mm. in diameter. The edge of the tumour is on the left. Near this is a zone of homogeneous intact tumour. On the right all the tissue is necrotic. In the intermediate zone are many prominent, dilated and congested blood vessels.

FIG. 15. Photomicrograph of tissue of tumour RIB5 from the intermediate zone shown in Fig. 16. What was homogeneous tissue has broken up into cylindrical systems of about 100 $\mu$ radius, with a dilated blood vessel at the centre. These blood vessels have walls of endothelial cells and basement membranes only. Surrounding them is a zone of intact tumour tissue. Outside this is a zone of necrotic tissue in which cells are clearly recognisable and in which the necrosis is relatively recent. Beyond this all the tissue is necrotic and details are lost. This necrosis is older.

1

3

Thomlinson.

14

15

Thomlinson.

*The Technique for Irradiation Tumours in Differing Pressures of Oxygen*

All irradiations were made with a Marconi Industrial Model X-ray set, with an electrically operated shutter, running at 250 kv. and 10 or 15 m.a. to give a dose rate of 400 rads per minute using filters of 0·5 mm. copper and 1·0 mm. aluminium. The mid-point of the tumour was approximately 21 cm. from the target of the X-ray tube. The dose given at each irradiation was controlled by using a monitor placed in front of the shutter. This consisted of an ionisation chamber through which the X-rays passed and the current of which was integrated. This current was correlated with the dose received at the position of the mid-point of the tumour as measured with a condenser-ionisation chamber. All the animals were treated within a spherical pressure chamber 9 inches in diameter

FIG. 2.—Diagram of the pressure chamber for treating rats at high pressures of oxygen, showing the mechanisms for controlling gas flow and temperature, the method of supporting and protecting the rat and the relation of the chamber to the monitor and X-ray tube.

made from ½ inch Perspex sheet moulded into two hemispheres with flanges, which were bolted together through a rubber gasket (Fig. 2 and 3).

The temperature within the chamber was kept at 30° C. ± 2° C. by passing the incoming gases either through a coiled pipe heated electrically or through a water cooled spiral. These alternative channels were controlled by electrically operated valves governed by a thermostat within the chamber. Mixing of gases in the chamber was ensured by a baffle just within the entrance port. When oxygen was passed into the air-filled chamber at 10 l./min. less than 0·5 per cent nitrogen, measured by a mass spectrometer, was present after two minutes. The gas pressure was regulated by a British Oxygen Company constant pressure delivery valve on the gas cylinders, and a needle valve near the chamber. This pressure was indicated by a pressure gauge on the chamber. The gas flow was regulated by a needle valve on the exit from the chamber with a gas flow-meter beyond this.

The anaesthetised rat was placed on its side on a couch in the upper part of the chamber so that the tumour lay free of any external support or traction.

This couch was attached to a wooden platform carried on a Perspex "spider" resting in turn in the lower hemisphere of the chamber. Also carried by this "spider", immediately above the rat, was a horizontal metal ring about the vertical axis of the chamber. In this ring could be placed one of several rings of lead, one inch in thickness, used to define the X-ray beam. This lead ring was therefore in a fixed position in relation to the lower hemisphere of the chamber and hence the upper hemisphere when that was in place. The upper hemisphere was brought into relation with the monitor on the outlet of the X-ray tube. The distance that the tumour lay from the X-ray tube was adjusted by a vertical movement of the couch and wooden platform in relation to the lead defining ring. A single measurement from the mid-point of the tumour to the under surface of the defining ring was all that needed to be made in calculating the monitor reading which indicated the selected dose.

It was possible by palpation to define the position of the deep surface of the tumour and the adjacent abdominal muscle and this was marked on the skin. With the animal lying on its side, the centre of the tumour was placed in the axis of the chamber in such a way that a verticle beam of X-rays was tangential to the abdominal musculature. A suitably sized defining ring was chosen to ensure irradiation of the whole tumour and protection of the intestine. The rest of the animal was covered by a sheet of lead rubber.

### *Procedure*

The rats were immobilised by anaesthesia produced with an intra-peritoneal injection of sodium amylobarbitone, 12·5 mg. for a 200 g. rat, and proportionately slightly less or slightly more with lighter or heavier animals.

When irradiation was to be given with the tumour anoxic a clamp made of two horse-shoe shaped pieces of Perspex was placed on the skin around the tumour, between it and the abdominal muscle and tightened to occlude the circulation. The animal was placed on the couch in the pressure chamber, which was then closed and air passed through it at a rate of about 1 litre per minute. The clamp was left in position for six minutes before radiation began and removed immediately after its completion.

For irradiation to be given with the animal breathing air sodium amylobarbitone was given in the same way and the rat placed in the pressure chamber with no interference to the tumour circulation. The chamber was closed and air passed through it. Irradiation was given immediately. On some occasions, after completion of irradiation, oxygen was given at pressure in the manner used for irradiation in oxygen.

When irradiation was given with the animal breathing oxygen at high pressure it was anaesthetised and placed in the chamber in the usual way. Oxygen was passed through the chamber at 10 l./min. to flush out the nitrogen. The pressure within the chamber was then raised at a rate of 10 lb./min. The earliest rise in pressure sometimes caused movement of the animal. This could be stopped or minimised by raising the pressure more slowly. The extent of any movement could be seen from the marks on the skin, and if need be the chamber was opened and the process started again. The pressure of oxygen was raised to 45 lb. per square inch (4 atmospheres absolute) and maintained for fifteen minutes before irradiation.

Immediately before and immediately after irradiation in each condition the position of the tumour was inspected and the respiration rate measured. During treatment the animal was observed visually and the thermometer, pressure gauge and gas flow meter could be seen.

After the completion of irradiation in oxygen the pressure was lowered slowly. After using pressures of 30 lb. per square inch five minutes appeared to be a long enough time, but after 45 lb. per square inch pressure thirty to forty minutes were required, if damage to lungs or nervous system were to be avoided.

### *The Design of the Experiment*

This experiment was designed to test the hypotheses that in an intact malignant tumour there are cells which are not fully oxygenated, that these, after aerobic irradiation, are capable of regenerating the tumour in its environment and that they can be influenced by the breathing of higher pressures of oxygen.

The effect of irradiating tumours was therefore tested at two doses, 2000 rads and 4000 rads, and under each of three conditions, (i) the tumour being made anoxic by stopping its circulation, (ii) the tumour " aerated " with the rat breathing air, and (iii) the tumour " oxygenated " by the rat breathing oxygen at 45 lb. per square inch pressure. Results were obtained by making daily measurements of the diameter of the tumour in each of the three dimensions with graduated calipers and taking the arithmetic mean of these three measurements.

In addition to these irradiation " treatments " six types of control " treatment " have been carried out. In the first, only daily measurements were made. In the second, no irradiation was given but the animals were given the anaesthetic. In the third, the anaesthetic was given and the circulation to the tumours was occluded for twenty minutes. In the fourth the anaesthetic was given and the animal placed in oxygen at 45 lb. per square inch pressure for twenty minutes. The fifth type of control treatment was surgical excision of the tumour performed to test the frequency of metastasis formation before the time of treatment. The sixth control treatment consisted of giving the rats oxygen at 45 lb. per square inch pressure for 20 minutes after irradiation in air or after irradiation of the tumour in the anoxic condition.

Measurement of the growing tumour could be made from diameters of about 5 mm. upward. All treatments were given when the tumours had attained a mean diameter of between 8 mm. and 10 mm. Each form of treatment was given a number and was allotted to the tumours from a table of random numbers as they reached this size. The pre-requisite conditions for any treatment to be given were that the tumour was solitary, smooth, rounded and mobile in the subcutaneous tissue and clinically not attached to skin or muscle.

### RESULTS

The results of this experiment are based on the treatment of 82 tumours. Of these two animals were lost by death from haemorrhage into the lungs immediately after decompression in oxygen, when this had been done too quickly. A third animal having a doubtful additional minute nodule outside the field of radiation was discarded when this grew within a few days of treatment. A fourth was discarded from the surgical series because of local recurrence within one week.

TABLE I

| Type of treatment | Number of animals | Mean diameter of tumours (mm.) |
|---|---|---|
| Untreated control | 8 | 9·4 |
| Control given amylobarbitone only | 3 | 8·8 |
| Control given amylobarbitone and clamping of circulation | 3 | 8·6 |
| Control given amylobarbitone and oxygen at 45 lb. per sq. in. pressure | 3 | 8·6 |
| Surgical excision | 10 | 9·1 |
| 2000 rads given to anoxic tumours | 6 | 9·5 |
| 2000 rads with rat breathing air at atmospheric pressure | 7 | 9·1 |
| 2000 rads with rat breathing oxygen at 45 lb./sq. in. | 9 | 9·0 |
| 4000 rads with tumour anoxic | 6 | 8·5 |
| 4000 rads with rat breathing air at atmospheric pressure | 9 | 9·0 |
| 4000 rads with rat breathing oxygen at 45 lb./sq. in. | 14 | 8·8 |

Of the remaining seventy-eight tumours, the distribution amongst the different forms of treatment and the mean diameter of each group at the time of treatment is shown in Table I.

It should be noted that whilst the numbers in some of the control groups are still small these can be added to in subsequent experiments. The larger number of those treated with 4000 rads given in oxygen is due to an additional six being treated in succession at the end of the experiment.

*Result of surgery*

In all 11 tumours were excised. In one animal there was local recurrence at the site of the operation. On post-mortem examination no metastases were found. One of the remaining ten animals died on the 50th day after excision. In this animal there was no sign of neoplasm at the site of operation or in the axillary, inguinal or iliac lymph nodes. However, there were massive metastases in the lungs, in the upper mediastinum and around the lower part of the pericardium and upper surface of the diaphragm. The remaining nine animals are well and without sign of desease after more than 120 days from the time of excision.

*Results of control and radiation treatments*

In this first experiment it has seemed worth presenting the curves relating the mean diameter of each tumour to the day before or after treatment (R day) to show the extent of the variation. These curves are shown in Fig. 4 to 11.

In the untreated control group (Fig. 4) all tumours grew at a nearly uniform rate. Of the six animals shown, two died probably from haemorrhage into the tumour, before a diameter of 50 mm. was reached. In the second control group (Fig. 5) the growth rate of all tumours, whether treated with anaesthetic only, anaesthetic and clamp or anaesthetic and oxygen, was similar and fell within the range of the untreated controls. After 2000 rads given with the tumour made anoxic (Fig. 6) the growth rates were similar and slightly but appreciably delayed as compared with the control. After 2000 rads given whilst the animal was breathing air with the tumour circulation unimpaired (Fig. 7) there was considerable delay in growth and more variation from tumour to tumour. This variation was more prominent after 2000 rads given with the rat breathing oxygen at 45 lb/square inch pressure (Fig. 8). One tumour of this group became impalpable after 29 days and the animal is well after 120 days. At the other extreme the growth rate of one tumour was similar to that of the anoxic group.

After 4000 rads given to the anoxic tumour (Fig. 9) there was once more less variation in response as compared with the air and oxygen groups and an appreciable slowing of growth as compared with 2000 rads given to the anoxic tumour. 4000 rads given with the rat breathing air (Fig. 10) resulted in some variation of

FIG. 4

response but there was a common trend of all but two of the nine tumours included. In the last group treated with 4000 rads whilst breathing oxygen (Fig. 11) the different response appeared to have resulted in two populations. One of these composed of four tumours had behaved like the anoxic group, whilst the other ten had followed a common trend, usually resulting in continued growth, but with two tumours disappearing for over 120 days up to the time of writing.

Possible reasons for these different responses will be discussed. Meanwhile the results of these different treatments may be compared from the means either of all the tumours in each group (Fig. 12) or, as seems more reasonable, those in each group which follow a common trend (Fig. 13). It will be noted how the variation within each group increases from the anoxic group through the air group to the oxygen group and is more marked with the higher dose than the lower.

The mean survival time of all animals in each group is shown in Table II.

Not all tumours grew after irradiation to reach the large size of 50 mm. in diameter. Three tumours were apparently cured. Many other animals died of

Fig. 5

TABLE II

| Treatment | Mean survival time in days |
|---|---|
| All controls . . . . . . . | 17±1·1 |
| 2000 rads, tumour anoxic . . . | 21±1·6 |
| 2000 rads, rat breathing air . . . | 28±1·3 |
| 2000 rads, rat breathing oxygen . . | 39±8·2 |
| 4000 rads, tumour anoxic . . . | 27±1·7 |
| 4000 rads, rat breathing air . . . | 37±0·8 |
| 4000 rads, rat breathing oxygen . . | 45±7·1 |

FIG. 6

metastases before the tumours had grown large. However, in each group several tumours have grown to 50 mm. in diameter, and the average times they have taken to do this are listed in Table III.

TABLE III

| Treatment | Time after treatment day for growth to 50 mm. diameter in days |
|---|---|
| Controls . . . . . . . | 14 (29 after implantation) |
| 2000 rads, tumour anoxic . . . | 18 |
| 2000 rads, rat breathing air . . . | 26 |
| 2000 rads, rat breathing oxygen . . | 36 |
| 4000 rads, tumour anoxic . . . | 26 |
| 4000 rads, rat breathing air . . . | 38 |
| 4000 rads, rat breathing oxygen . . | 46 |

No significant result has appeared from analysis of postmortem findings and these will not be presented in detail.

DISCUSSION

The object of the investigations of which this paper is a report is the elucidation of certain problems to do with the use of oxygen in combination with radio-

Tumour RIB5. 2000 rads. Rat breathing air.

Fig. 7

therapy in the treatment of human cancer. The first answer to be sought is a clear demonstration of the beneficial effect of oxygen with radiation in the treatment of a tumour irradiated and followed *in situ* in the host animal with no further interference than the making of daily measurements. Other practical questions such as the optimal pressure of oxygen which should be breathed, the minimal time for which oxygen should be given before treatment, the whole complex issue of the fractionation of the total dose, and the dose itself in relation to tumour size and type are awaiting answers. Clearly these will only come in the light of understanding of the radiobiology of neoplastic cells, the pathology of tumour growth and the physiology of oxygen transport in the animal body and within the tumour itself.

FIG. 8

*The pathological basis of the experiments*

Radiobiological aspects of the effect of oxygen on systems of cells, neoplastic and otherwise have been extensively studied and recently reviewed (Alper, 1960; Gray, 1958; Scott, 1958). It seems probable that all nucleated cells will show a similar relationship between oxygen availability at the time of irradiation and sensitivity to radiation injury as judged by damage to the reproductive integrity of the cells. Similarity between the cells of the experimental sarcoma RIB5 used in the experiments reported here and, for example, the Ehrlich mouse ascites tumour (Deschner and Gray, 1959) has been assumed, but must be confirmed in due course.

Direct evidence of the presence of cells at low concentrations of oxygen in intact tumours at the time of irradiation is scanty and uncertain. The probable existence of oxygen gradients in certain types of carcinoma such as would be likely to cause low levels of oxygen availability to some cells has been suggested

FIG. 9

(Thomlinson and Gray, 1955). The type of diffusion pattern which they reasoned must exist was considered in relation to the circulation and necrosis in other types of tumour by Churchill-Davidson, Sanger and Thomlinson (1957).

The importance of this relationship lies in the frequency with which a common pattern of vascular disturbance and necrosis is found in most types of malignant growth including human cancer. For example, when tumour RIB5 has grown to a large size, say 30 mm. or more in diameter, the whole centre has undergone coagulative necrosis and at the periphery is an irregular zone of intact tumour tissue ranging from 5 mm. to 10 mm. in thickness. In the outer part of this zone there is no necrosis. A little towards the centre, small areas of necrosis appear at points at the greatest distance from blood vessels. In the region bordering on the necrotic centre the intact cells appear to break up into cylindrical systems surrounding dilated and congested blood vessels, the walls of which are of capillary structure (Fig. 14 and 15). At the periphery of these systems are zones of necrotic

Fig. 10

cells where cell membranes are yet intact and cell structure is visible. At greater distances from the central capillary the whole tissue is necrotic and no cellular detail is visible. In a map of such a region (Fig. 16) the uniformity of width of intact cells around each capillary and the regularity of the zone of necrotic but recognisable cells suggests a dynamic system in which there is a gradual shrinking of the amount of tissue nourished by each capillary. This in turn suggests a gradual progressive failure of the circulation within these capillaries. It is hard to believe that there are not gradients of oxygen tension falling with distance from such capillaries. It seems reasonable to suppose that living cells in these tumours which are in low concentrations of oxygen, if they exist, are to be found at the greatest distances from such capillaries, that is to say, on the borders of necrotic areas.

FIG. 11

If this picture of the presence and position of hypoxic cells in a tumour is right, it seems that in the natural course of events their " days are numbered " and it may be supposed that they are of no concern to the radiotherapist since they cannot become " more dead than dead " (Bevan). However, it is a hypothesis that after irradiation in aerobic conditions these cells may survive because of the protective effect of hypoxia and regenerate the tumour in the better nutritional conditions which follow the death and absorption ot their more radiosensitive neighbours.

John's hypothesis (see Churchill-Davidson, Sanger and Thomlinson, 1957) suggests the massive necrosis in tumours is due to venous infarction following slowly progressive venous obstruction consequent upon the expansion of the tumour mass in a limited space. If this were correct it would imply that more oxygen

FIG. 12.—The arithmetic means of the growth curves of all tumours in each group. Increased oxygen tension, from anoxic conditions through breathing air to breathing oxygen, results in increased damage and delayed growth of the tumour with each dose. Some curves stop earlier than the 30th day because of the death of a few animals with large tumours. Note the matching of the curves after 4000 rads given to the anoxic tumour and 2000 rads in air and those of 4000 rads in air and 2000 rads in oxygen.

can only reach regions in which it is deficient if each volume of blood were to carry more, since further vasodilation would not increase the blood-flow. This was the basis upon which the breathing of oxygen at high-pressures was introduced at St. Thomas's Hospital.

In developing an experimental system to study the use of oxygen it seemed essential to use a neoplasm which grew to form a pattern of necrosis and vascular change similar to that found in human neoplasms and also which extended by infiltration and metastasis formation. The latter is important because it is at least possible that the profound inflammatory changes following irradiation may influence the spread of the disease by this means.

A transplantable tumour is the only type of neoplasm which permits these factors to be studied quantitatively. The objections to the use of transplantable

FIG. 13. The arithmetic means of the growth curves of those tumours in each group following a common trend. The standard errors of the means are shown. Note the match between the curves after 4000 rads given to the anoxic tumour and 2000 rads given in air and those of 4000 rads given in air and 2000 rads in oxygen.

tumours lie in the realms of tumour immunology. It may well be that no such tumour is isogenic with its host (Prehn and Main, 1957). However, the influence of immunity can be minimised by using in-bred strains of animal and tumours arising spontaneously or by induction within the strain. The degree of incompatibility can be tested by using the cell dilution techniques of Hewitt (1958) and the four tests suggested by Scott (1960) are being carried out. The effect of immunological incompatibility between tumour and host is to produce cures after irradiation when cells have survived the radiation injury in such numbers as would have regenerated the tumour if no immune reaction existed. The relations between radiation damage and immunological response are very complex and have recently been discussed (Scott, 1960). These inter-relations make the exact comparison of the groups in these experiments rather difficult but the possible effects of immunity are diminished when comparison is made between dose and oxygen concentration which produce equal damage to the tumour.

*Transplantation technique*

The development of a technique which would admit this type of comparison has proved surprisingly difficult. At first, small masses of apparently healthy solid tumour were implanted subcutaneously, but these resulted in irregular

FIG. 16.—A diagrammatic map of tissue in the intermediate zone as shown in Fig. 14 and 15. The uniform width of the zone of " recent necrosis " suggests a dynamic system with gradually failing circulation in the " capillaries ".

shaped tumours which frequently grew into skin or muscle. Centrifuged free cell suspensions were then injected into subcutaneous tissue through a fine hypodermic needle, but these tumour cells followed the needle track and the incidence of early lymph node metastases was high.

At this stage a mixture of a centrifuged cell suspension and sodium alginate was first used in proportions of two parts to one. This mixture was dropped into a 1 per cent solution of calcium chloride and left in it for varing lengths of time. It had been determined that the tumour cells alone would grow after ten minutes immersion in this solution. It was found that after 20 seconds immersion the calcium alginate formed at the periphery of a drop of the mixture produced a capsule such that the tumour pellet could be handled by pipette and be implanted in the subcutaneous tissue. However, whilst almost all these " pellets " grew to form tumours many of these followed the needle track and infiltrated the skin. The impression was formed that the " pellets " were too fragile and ruptured after implantation. At the other extreme " pellets " left in calcium chloride for five minutes were almost solid and failed to grow at all. Eventually a time of $1\frac{1}{4}$ minutes was selected as giving a reasonable yield of tumours (about 75 per cent of those implanted).

Irradiation experiments carried out with those tumours resulted in a disappointing loss of usable material within a few days following treatment either from the death of animals due to metastases or the rupture of tumours through the skin.

An extensive series of surgical excisions of the primary tumours at varying sizes from 3 mm. to 12 mm. in diameter resulted in 50 per cent cures irrespective of size. This indicated that metastasis formation, or spread of the tumour beyond the field of excision and therefore of irradiation, had occurred in the other 50 per cent of cases as a result of the transplantation technique. Experiments starting from this situation seemed fruitless.

During the course of these experiments it was realised that those tumours which, at the size of 10 mm. diameter, felt smooth and rounded, and which were in the clinical sense mobile in the subcutaneous tissue and unattached to skin or muscle, went on to grow to a large size without obvious metastases before a late stage in the disease. This seemed to be the condition at the 10 mm. size in which the tumours were suitable for experiment. The problem appeared to be to hold the transplanted cells together in a mass until the trauma produced by the transplantation process had healed, or at any rate until a barrier had formed around the transplant.

Recollecting, from the days of keeping pigs during the war, the way of making sausage skins from small intestine, it was decided to try to make viable tumour " sausages ". In the first place the viability of tumour cells within a capsule of intestinal wall was tested by scraping off the mucosa of the adult rat jejunum and filling the lumen with minced tumour. This was then tied into suitable lengths and implanted. The tumours all grew and histological examination showed the presence, not only of viable cells, but of newly formed blood vessels both within and without the remnants of the intestinal wall. In spite of washing and searing it is quite possible that many viable tumour cells were left beyond the " ties " at the end of each sausage, and therefore not surprising that the resultant tumours were of irregular shape. The next step, however, yielded satisfactory results. This was to implant fragile alginate tumour pellets into the lumen of the inverted intestine of a young rat. The pellets held the tumour cells together until the ties had been made. These spherical " sausages " have given a good yield of tumours meeting the conditions required for experiment. A few have infiltrated muscle but this was probably due to implantation in too deep a subcutaneous layer. The results of the small series of surgical excisions performed so far indicate that at the 8 mm. to 10 mm. size the chances of the tumour being still localised are high. It seems likely that this technique can be used for the implantation of other types of tumour and will enable *in vivo* comparisons to be made of other types of cancer therapy as well as radiation.

*Irraduation technique*

Little comment need be made on the radiation technique except to emphasise the necessity of avoiding any manipulations which may impair the circulation in the tumour, bearing in mind the fragile structure and the low intravascular pressures of the veins. Any question of interference with these invalidates the results of experiments involving oxygenation. Variations of skin circulation with environmental temperature should also be borne in mind.

The need to reduce the pressure in the chamber very slowly after irradiation in oxygen became apparent when two animals developed spastic paraplegia of the upper limbs. This was probably caused by some embolic phenomenon in the spinal cord and although there has been recovery, it is not complete.

The apparatus used in these experiments is being modified to demonstrate radiographically the presence of the whole tumour in the radiation field immediately before and after the dose is given.

*Results*

The most disturbing feature in the results presented from this first experiment is the scatter of the growth curves following irradiation in air and more still in oxygen. This is more obvious with the higher dose and most pronounced in the separation into two populations seen after 4000 rads. given in oxygen. Although this variability is explicable in terms of small differences in the number of cells surviving radiation the different response may come to reflect the difference between cure and failure in treatment. Five possible explanations may be advanced.

First, that the tumours growing more rapidly after irradiation in fact contained a rather larger number of viable cells at the time of irradiation. Provided that all the cells are well oxygenated, where two out of fourteen tumours have been completely killed by 4000 rads, all would be expected to be killed by 5000 rads. The effect of the small possible difference in numbers at the time of irradiation would disappear in this case, and the result of experiment with a higher dose will decide this explanation.

Second, that the whole tumour was not irradiated. Whilst this seems unlikely, steps are being taken to demonstrate radiographically that the whole of each tumour is in the radiation field at the beginning and end of treatment.

Third, that parts of the tumour remained hypoxic in spite of oxygen administration. This seems the most likely explanation and quantitative predictions of the effect of the presence of a few anoxic cells are consistent with these results. (Hewitt, 1959). The use of different pressures of oxygen may resolve this. It is possible that damage to the lungs from breathing oxygen (Bean, 1945) might have impaired the oxygenation of the arterial blood, either because of pulmonary oedema or intrapulmonary shunting of blood. However, local hypoxia in the tumour seems a more likely explanation than general arterial hypoxia.

Finally, a radioresistant strain of cells can be postulated, but this seems unlikely (Conger, 1956 ; Nice, 1957).

The requirement of the experiment to produce matching results with differing doses of irradiation and conditions of oxygenation has been approximately achieved. Whether comparison is made between all tumours in each group (Fig. 12) or those in each group following a common growth pattern (Fig. 13) two pairs of growth curves are nearly the same. The effect of 4000 rads given to the anoxic tumour is roughly equivalent to the effect of 2000 rads given to the tumour with the rat breathing air. The effect of 4000 rads given to the tumour with the rat breathing air and 2000 rads to the tumour with the rat breathing oxygen at 45 lb/square inch pressure are nearly equal. Also the difference between one pair and the other is statistically significant.

Since the whole growth pattern of the tumours has been disturbed by any of these forms of radiation, it may be doubted that the matchings of one growth curve with another at any particular point is valid. Very good matches could be obtained between all the curves within the first five post-irradiation days! It therefore seems worth while to consider and later to investigate the factors which govern the shape of any of these curves.

Clearly the curves are composite, representing on the one hand the rate of removal of dead tumour tissue, and on the other the multiplication of surviving tumour cells. The curve of the rate of removal of dead tissue will also be composite, because dead cells lying amongst capillaries with an active circulation are eliminated very much more rapidly than a necrotic mass which has to be reorganized. Both these processes are likely to be affected by the effects of radiation on the capillaries.

A number of possibilities affect the shape of the curve representing multiplication of the tumour cells surviving radiation.

The linear relationship between the radius of the tumour and time in the control curve suggests that cell death is taking place in the larger tumours at about the same rate as cell production. In these tumours the whole central region is necrotic and is surrounded by a viable rim. If this rim maintains a constant thickness, which approximately it does, its volume increases five-fold as the radius of the tumour doubles. Since the time taken to double the radius is about four and a half days and the number of intact tumour cells is proportional to the volume of the rim, the generation time of these cells is slightly less than one day.

The curve of tumour size after irradiation with 4000 rads in oxygen shows a doubling of the radius in twelve days, beginning on the fourteenth post-irradiation day. This might indicate a generation time of two and a half days—an unlikely delay of metabolic processes in cells surviving the first two weeks. However, the growth rate may be reduced by nutritional deficiency due to the effects of radiation on capillary blood-vessels. Another possibility is that many cells have suffered less than lethal genetic damage and that in a series of cell divisions damaged material is gradually eliminated in non-viable daughter cells. In this way the total number of viable cells and therefore the tumour mass might remain almost constant for a long period.

In the animals which survive long enough for the tumour to reach the large size of 50 mm. diameter the growth curves gradually steepen and come almost or quite equal to the slope of the control group. The times taken to reach this size are shown in Table III. The longest time was 46 days after the dose of 4000 rads in oxygen. This may be compared with the control group which reached this size 14 days after the treatment day and 29 days after implantation. The difference between 29 days and 46 days might be explicable in terms of the number of cells surviving the implantation process in the first case and the number surviving irradiation in the second. If this is so, the shape of curves representing the cell multiplication processes could be identical and the times taken by each group to reach the large size would be proportional to the number of cells surviving. It is interesting that on this basis there is also a close match between the group receiving 4000 rads in anoxic conditions and 2000 rads in air, and the group receiving 4000 rads in air and 2000 rads in oxygen. It will be of interest to investigate the various possible factors influencing the shape of the curves.

These results confirm those of earlier workers with " solid " tumours (Holcroft,

Lorenz and Matthews, 1952; Scott, 1953; Dittrich and Stuhlmann, 1954; Grüssner, 1957; du Sault, Eyler and Dobben, 1959). Whilst no great mathematical precision should be attached to the ratio of 4 : 1 shown in the effect on the tumour with the rat breathing oxygen compared with the anoxic tumour the results do support three conclusions :

1. In the tumour RIB5 there are cells which are protected by anoxia from radiation damage whilst the animal is breathing air.
2. After irradiation "in air" these cells are capable of multiplying to regenerate the tumours, and
3. The radiation injury to these cells is enhanced by giving the rat oxygen to breathe at 45 lb. pressure.

The physiological and pathological mechanisms bringing about these effects are equally likely to apply in human tumours as in rat tumours where the same patterns of growth and circulatory disturbance are found. It is therefore likely that the use of oxygen in the radiotherapy of human cancer will diminish the number of cancer cells surviving a given dose of radiation and increase the proportion of patients cured.

SUMMARY

A technique has been developed for growing transplantable malignant tumours in the subcutaneous tissue of the rat in such a way that they remain localised until they have grown to a suitable size—10 mm. diameter—for testing the effects of different treatments. The course of the tumours was followed *in situ* by daily measurement. Comparisons have been made of the effects of single doses of 2000 rads and 4000 rads of 250 kv. X-rays under three different conditions of oxygenation of the tumour; with the tumour made anoxic by clamping the circulation, with the tumour "aerated" with its circulation intact and the rat breathing air at atmospheric pressure and with the tumour "oxygenated" with the animal breathing oxygen at 4 atmosphere's pressure. The effect of 2000 rads given in air approximately equals that of 4000 rads to the anoxic tumour and the effect of 2000 rads in oxygen approximately equals that of 4000 rads in air. These results indicate that when the rat breathes air there are cells in the tumour protected from radiation injury by hypoxia; that after radiation in air such cells can regenerate the tumour and their radiosensitivity can be enhanced by the breathing of oxygen at high pressures. The pathological basis of these conclusions suggests that they apply equally to many forms of human cancer.

I should like to thank Sir Robert Davis for the gift of the pressure chamber used in these experiments, Dr. J. B. West of the Post Graduate Medical School for measurements with the mass spectrometer, Mr. D. Moore for measurements of radiation dose, my technician Mr. J. Whitaker, and other members of this Unit, almost all of whom have helped to make the work possible.

### REFERENCES

Alper, T.—(1960) *Annu. Rev. nuclear Sci.*, 1960. In press.
Bean, J. W.—(1945) *Physiol. Rev.*, **25**, 1.
Churchill-Davidson, I., Sanger, C. and Thomlinson, R. H.—(1955) *Lancet*, i, 1091.
—(1957) *Brit. J. Radiol.*, **30**, 406.
Conger, A. D.—(1956) *Radiology*, **66**, 63.

DESCHNER, E. E. AND GRAY, L. H.—(1959) *Radiation Res.*, **11**, 115.
DITTRICH, W. AND STUHLMANN, H.—(1954) *Naturwissenschaften*, **41**, 122.
GRAY, L. H.—(1958) ' Lectures on the Scientific Basis of Medicine '. Vol. VII, 1957–58, p. 314.
*Idem*, CONGER, A. D., EBERT, M., HORNSEY, S. AND SCOTT, O. C. A.—(1953) *Brit. J. Radiol.*, **26**, 638.
GRÜSSNER, G.—(1957) *Strahlentherapie*, **104**, 514.
HEWITT, H. B.—(1958) *Brit. J. Cancer*, **12**, 378.—(1959) *Ibid.*, **13**, 675.
HOLCROFT, J. W., LORENZ, E. AND MATTHEWS, M.—(1952) *J. nat. Cancer Inst.*, **12**, 751.
NICE, C. M.—(1957) *Amer. J. Roentgenol.*, **78**, 831.
PREHN, R. T. AND MAIN, J. M.—(1957) *J. nat. Cancer Inst.*, **18**, 769.
SCOTT, O. C. A.—(1953) *In* Gray, Conger, Ebert, Hornsey and Scott. *q.v.*—(1958) ' Advances in Biological & Medical Physics ', Vol. 6. New York (Academic Press).—(1960) *Radiation Res.* In press.
DU SAULT, L. A., EYLER, W. R. AND DOBBEN, G. D.—(1959) *Amer. J. Roentgenol.*, **82**, 688.
THOMLINSON, R. H. AND GRAY, L. H.—(1955) *Brit. J. Cancer*, **9**, 539.